# REMI: Reconstructing Episodic Memory During Intrinsic Path Planning

**DOI:** 10.1101/2025.07.02.662824

**Published:** 2025-07-03

**Authors:** Zhaoze Wang, Genela Morris, Dori Derdikman, Pratik Chaudhari, Vijay Balasubramanian

**Affiliations:** 1 Department of Electrical and Systems Engineering, University of Pennsylvania, Philadelphia, PA 19104, USA; 2 Tel Aviv Sourasky Medical Center, Tel Aviv 6423906, Israel; 3 Gray Faculty of Medical and Health Sciences, Tel Aviv University, Tel Aviv 6997801, Israel; 4 Rappaport Faculty of Medicine, Technion – Israel Institute of Technology, Haifa 31096, Israel; 5 Department of Physics, University of Pennsylvania, Philadelphia, PA 19104, USA; 6 Rudolf Peierls Centre for Theoretical Physics, University of Oxford, Oxford OX1 3PU, UK; 7 Santa Fe Institute, Santa Fe, NM 87501, USA

## Abstract

Grid cells in the medial entorhinal cortex (MEC) are believed to path integrate speed and direction signals to activate at triangular grids of locations in an environment, thus implementing a population code for position. In parallel, place cells in the hippocampus (HC) fire at spatially confined locations, with selectivity tuned not only to allocentric position but also to environmental contexts, such as sensory cues. Although grid and place cells both encode spatial information and support memory for multiple locations, why animals maintain two such representations remains unclear. Noting that place representations seem to have other functional roles in intrinsically motivated tasks such as recalling locations from sensory cues, we propose that animals maintain grid and place representations together to support planning. Specifically, we posit that place cells auto-associate not only sensory information relayed from the MEC but also grid cell patterns, enabling recall of goal location grid patterns from sensory and motivational cues, permitting subsequent planning with only grid representations. We extend a previous theoretical framework for grid-cell-based planning and show that local transition rules can generalize to long-distance path forecasting. We further show that a planning network can sequentially update grid cell states toward the goal. During this process, intermediate grid activity can trigger place cell pattern completion, reconstructing experiences along the planned path. We demonstrate all these effects using a single-layer RNN that simultaneously models the HC-MEC loop and the planning subnetwork. We show that such recurrent mechanisms for grid cell-based planning, with goal recall driven by the place system, make several characteristic, testable predictions.

## 1 Introduction

Mammals employ various cell types to represent space and guide navigation [1–3]. For example, grid cells (GCs) in the Medial Entorhinal Cortex (MEC) fire in hexagonally arranged patterns as an animal moves through an environment [4–6]. Grid cells also exhibit multiple spatial scales in their periodic firing patterns [7–10], which may arise through self-organization driven by inhibitory gradients in MEC attractor networks [11], enabling the efficient encoding of a large number of locations [12]. Meanwhile, hippocampal place cells (HPCs) fire at selective locations within an environment in a seemingly random manner [1, 2]. However, they are tuned not only to spatial locations but also to experiences and contextual cues [13–17]. The HPC population responses remap to form distinct representations in different environments and reinstate prior maps in familiar contexts. This ability supports their capacity to encode multiple environments without interference [2, 13, 18–20].

The local circuitry driving single GC and HPC activity has been studied extensively, and recent work shows that many of the phenomena they manifest can emerge in trained neural networks modeling their functional roles. Path integration theories propose that GCs track an animal’s position by integrating its movement, forming a continuous attractor landscape [4, 21, 22]. Likewise, recurrent neural networks (RNNs) trained to infer position from movement sequences develop grid-like patterns in their hidden layers [23–27]. For place cells, one view is that they could be emergent patterns that arise from encoding sequences of sensory signals relayed partly through the MEC [28–30] during spatial traversal [15–17, 31], perhaps implemented by a continuous attractor network (CAN) [32–35]. An alternative account is the successor representation framework [36, 37], which suggests that HPCs may encode predictive representations of future spatial states [38, 39].

Building on extensive studies of the emergence and functional roles of grid and place cells [15–17, 23, 25, 27, 40, 41], a natural view is that animals maintain these representations not just to encode space, but to support intrinsically motivated navigation tasks such as recalling goal locations from partial cues and planning paths to them. This view is supported by evidence that during rest or sleep, the hippocampus reactivates neural sequences that either mirror past paths (replay) or form novel trajectories through unvisited locations (preplay) [42, 43]. Yet, theories of place cell emergence suggest their dynamics are updated by sensory input sequences. Any planning network driving replay or preplay must therefore reproduce these inputs, or at least the sequence of sensory latent representations along the trajectory. However, if such a network could directly store and recall detailed sensory information along trajectories, the role of place cells in recall would be redundant, contradicting their well-established importance in spatial memory

One candidate framework suggests that predictive information encoded by place cells supports transitions from the current location to neighboring states. This encoded transition likelihood traces out a most likely path connecting the current and goal locations [37]. However, this framework requires the animal to visit and encode all locations in the environment, and therefore cannot explain how animals might take shortcuts through unvisited locations. An alternative idea leverages residue number systems, enabled by multiple scales of grid cells, to encode pairwise displacements [44]. However, while grid cells can represent a vast number of locations, encoding pairwise relations among them quickly becomes intractable, fails to account for hippocampal replay and preplay, and does not explain how grid patterns would be recalled to support navigation.

Inspired by recent work suggesting that place cells may autoassociatively encode sensory inputs [16], or more generally, signals weakly modulated by space, we propose a framework unifying grid-cell-based planning with contextual recall in the hippocampus. The spatial stability of grid representations [45] allows us to treat grid cell activity as a special form of observation derived from self-motion and modulated by space. Given that the MEC, which projects to the hippocampus, contains both grid cells and neurons relaying diverse sensory signals [28–30], we propose that if place cells autoassociate both, then: (1) partial sensory input will reactivate the corresponding grid cell state through hippocampal autoassociation, enabled by recurrent connections between HC and MEC; (2) a planning network operating in grid space can use stable relative phase relationships across rooms to plan paths without rewiring when entering new environments; (3) the known local transition rules of grid cells will enable the network to construct valid paths even through unvisited areas; and (4) during planning, grid representations will advance to intermediate states, and the hippocampus will retrieve the associated sensory cues, enabling reconstruction of sensory experience along the planned trajectory.

## 2 Method

### 2.1 Hippocampus–Medial Entorhinal Cortex Loop

During spatial traversal, animals form internal representations of the world shaped by olfactory, visual, and auditory cues. These sensory signals are partially reflected by (weakly) spatially modulated cells (SMCs) in the MEC [28] and relayed to the hippocampus (HC) [46, 47]. Such observations signal the presence of, e.g., food, water, or hazards, and thus may serve not only as inputs but also as objectives for navigation. Animals also receive displacement signals from the vestibular system, proprioception, and visual flow. Although these signals may not directly correspond to specific objectives, they help animals infer their relative position from previous locations. This process is supported by grid cells (GCs) in the MEC through path integration [4, 22, 48, 49].

However, both sources of information can fail during navigation. Sensory observations may be unavailable in conditions such as dim light or nighttime, while path integration may break down due to loss or corruption of speed or direction signals. We hypothesize that animals can exploit spatial correlations between these two sources of information to achieve more accurate localization than either source alone. However, given that these correlations are context-dependent, for example varying across compartments of an enclosure or configurations of natural landmarks, this relationship is unlikely to be supported by fixed wiring between SMCs and GCs within the MEC. Instead, we propose that hippocampal place cells (HPCs) encode these correlations through pattern completion, allowing the two types of information to be flexibly wired across contexts.

With this coupling between SMCs and GCs through HPCs, animals can directly retrieve a location’s GC representation from sensory cues. The animal can then use this recalled GC representation to plan a path, as planning with GC representations is not context dependent and can therefore be generalized to any environment [44]. Along a GC-planned path, this coupling could, in turn, reconstruct SMC activity from intermediate GC states. Since GCs maintain stable relative phase relationships across environments, we further propose that planning based solely on GC representations enables shortcuts through unvisited locations and immediate planning when re-entering familiar rooms. These capabilities are not supported by HPCs alone, as they encode discrete locations and lack the continuous spatial structure required for planning. To test this proposal, we construct an RNN model of the HC-MEC loop and build a planning model on top of it.

#### 2.1.1 A RNN Model of the HC-MEC Loop

##### Integrate input and output cells directly into recurrent dynamics.

Previous RNN models characterizing emergence of GCs and HPCs [16, 23, 25, 27, 50] have a limitation for planning: they typically do not explicitly model signals driving recurrent dynamics but instead rely on a learnable projection matrix. We posit that, during planning, these signals serve as control inputs to drive the HC-MEC loop toward the goal (see Sec. 4). Explicitly modeling these inputs simplifies the architecture and enables joint modeling of HC, MEC, and the planning subnetwork within a single recurrent structure.

Consider a standard RNN used in previous studies [16, 23, 25, 27, 50], updating its dynamics as:

(1) $$z_{t+1}=\alpha \cdot z_{t}+(1-\alpha )\cdot \left(W^{in}u_{t}+W^{rec}\;f\left(z_{t-1}\right)\right)$$

where $z\in {\mathbb{R}}^{d_{z}}$ is the hidden state, *u* is the input, *α* is the forgetting rate, and **W**^*in*^, **W**^*rec*^ are the input and recurrent weight matrices. The output is given by a linear readout: $y_{t}=W^{\text{out}}z_{t}\in {\mathbb{R}}^{d_{o}}$. We extend this RNN by introducing auxiliary input and output nodes, *z^I^* and *z^O^*, and update as:

(2) $$\left[\begin{array}{l} z_{t+1}\\ z_{t+1}^{I}\\ z_{t+1}^{O} \end{array}\right]=\alpha ⊙\left[\begin{array}{c} z_{t}\\ z_{t}^{I}\\ z_{t}^{O} \end{array}\right]+(1-\alpha )⊙\left(\left[\begin{array}{c} 0\\ u_{t}\\ 0 \end{array}\right]+\left[\begin{array}{ccc} W^{\mathrm{rec}} & {\tilde{W}}^{\mathrm{in}} & W^{\left(13\right)}\\ W^{\left(21\right)} & W^{\left(22\right)} & W^{\left(23\right)}\\ {\tilde{W}}^{\mathrm{out}} & W^{\left(32\right)} & W^{\left(33\right)} \end{array}\right]\;f\left(\left[\begin{array}{c} z_{t}\\ z_{t}^{I}\\ z_{t}^{O} \end{array}\right]\right)\right)$$

Here, *z^I^* directly integrates the input *u_t_* without a learnable projection, while *z^O^* is probed and supervised to match simulated ground-truth cell responses. This design eliminates the need for projection matrices **W**^*in*^ and **W**^*out*^. Instead, ${\tilde{W}}^{\mathrm{in}}$ and ${\tilde{W}}^{\mathrm{out}}$ act as surrogate mappings for the original projections. We set *α* as a learnable vector in ${\mathbb{R}}^{d_{z}+d_{I}+d_{O}}$ to allow different cells to have distinct forgetting rates, with ⊙ denoting element-wise multiplication.

We assign speed cells and allocentric direction cells as input nodes that only receive inputs. The SMCs are set to both input and output nodes, trained to match the simulated ground truth. These SMCs are assumed to primarily respond to sensory cues during physical traversal. Supervision constrains their dynamics to reflect tuning to these signals, while learned recurrent connections formed during training are intended to reflect Hebb-like updates in brain that preserve this tuning structure. The ground truth signals for all cells are strictly positive to reflect firing rates (see Suppl. 3 for how ground truth signals are simulated). For direction cells, we assign allocentric preferred directions uniformly over [0, 2*π*) with a fixed angular tuning width, ensuring their responses remain non-negative and their population activity lies on a 1D ring.

##### Partially Supervise Grid Cells.

For our proposed planning mechanism, we must model a complete HC-MEC loop containing stable patterns of both GCs and HPCs. However, no existing model exhibits the simultaneous emergence of grid and place cells. To address this, we supervise GCs to learn path integration. Specifically, we simulate GC population responses within a room and use them as ground truth. At the start of each training trajectory, RNN hidden units modeling grid cells are initialized to the ground-truth GC responses at the starting location. Along this trajectory, the network receives only speed and direction signals, directly input to the corresponding cells and relayed to the GC subpopulation through recurrent projections. We collect GC subpopulation states over time and penalize their deviation from simulated ground-truth responses, encouraging controllable GC activity while learning path integration. We refer to this as ***partial supervision*** and apply it to GCs for three reasons: (1) our primary focus is to test planning by the HC-MEC loop rather than the co-emergence of both GCs and HPCs; (2) HPC remapping in novel environments involves extensive reorganization of spatial tuning and synaptic connections driven by sensory input [40, 51, 52], making it harder to parameterize and simulate than GCs, which exhibit greater stability [45] and maintain stable relative phase relationships across environments [3, 53]; (3) the planning mechanism critically depends on the experimentally established stability of GC phase relationships and multiple spatial scales, as we will show in the following sections; these properties are difficult to control in existing emergence models.

We recognize that, biologically, HPCs appear before grid cells (GCs) [3, 54–56]; however, HPC firing fields become more spatially precise as GCs mature, suggesting an iterative refinement process [56, 57]. Conceptually, our HC-MEC model with partially supervised GCs can be seen as capturing this refinement phase, where the emergence of GCs enhances the spatial specificity of HPCs. Reflecting this process, we observe a quick emergence during early training and a corresponding reduction in HPC firing field widths after GCs are learned (see Suppl. 5.1).

##### Testing GC Path Integration and HPC Emergence.

**Figure 1:**
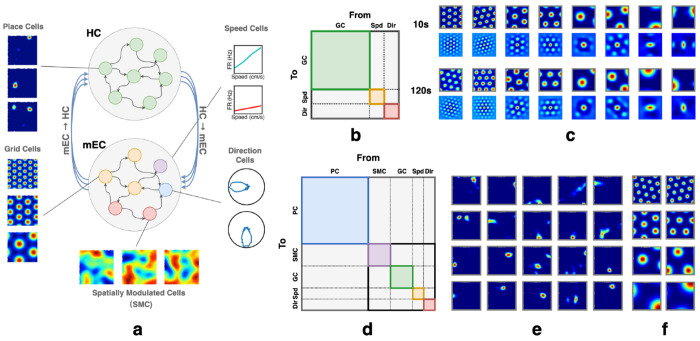
**(a)** RNN model of the HC-MEC loop. The top subnetwork contains HPCs, with example emergent place fields shown on the left. The bottom subnetwork includes partially supervised GCs, as well as supervised speed, direction, and spatially modulated cells (SMCs); example grid fields shown at left. **(b)** & **(d)** Speed cells and direction cells are denoted as *Spd* and *Dir*, respectively. Colored regions highlight within-group recurrent connectivity to indicate the partitioning of the connectivity matrix by cell groups. However, at initialization, no structural constraints are enforced. The full connectivity matrix is randomly initialized. **(b)** Illustration of the path-integration network’s connectivity matrix. **(c)** The network is trained to path-integrate 5s trials and tested on 10s trials (*L* = 100.50 ± 8.49 cm); the grid fields remain stable even in trials up to 120s (*L* = 1207.89 ± 30.98 cm). For each subpanel (10s, 120s): top row shows firing fields; bottom row shows corresponding autocorrelograms. **(d)** Illustration of RNN connectivity matrix of full HC-MEC loop. **(e-f)** Example place fields (emergent) and grid fields in the full HC-MEC RNN model.

Planning requires stable GC and/or PC representations. Before discussing planning, we first train the network to develop these representations. We first test whether a GC network trained with partial supervision can perform path integration. We simulate six modules with spatial periodicities scaled by the theoretical optimal factor $\sqrt{e}$ [12]. The smallest grid spacing is set to 30 cm, defined as the distance between two firing centers of a grid cell [58]. The grid spacing to field size ratio is 3.26 [59], with firing fields modeled as Gaussian blobs with radii equal to two standard deviations. We train this model on short random trajectories (5 s) but accurately path-integrate over significantly longer trajectories (10 s, 120 s) during testing (Figure 1c).

We then test the full HC-MEC loop model. Both supervised and partially supervised cells receive masked input and initial states, with supervised cells additionally receiving inputs at all timesteps. The HPC subnetwork receives signals only from MEC through recurrent connections and does not take external input. All cells are modeled within a single recurrent network without explicit connectivity constraints (see Figure 1d for initialization details). We observe HPCs emerge in the network, while GCs learn to path integrate (Figure 1e–f).

## 3 Recalling MEC Representations from Sensory Observations

We first tested that sensory observations at the goal location can trigger retrieval of the corresponding grid cell (GC) representation through auto-association of GCs and spatially modulated cells (SMCs). Auto-association is expected to occur when the input pattern is incomplete or degraded. To evaluate this, we trained nine models with identical configurations, varying only the masking ratio *r*_mask_ from 0.1 to 0.9. The masking ratio *r*_mask_ specifies the maximum fraction of direction, speed, GC, and SMC inputs, as well as their initial hidden states, that are randomly set to zero during training. This simulates degraded observations and encourages the network to learn robust recall through auto-association. Each model was trained on randomly sampled short trajectories using a fixed *r*_mask_, with new random masks generated for every trajectory and varying across time and cells. Masks were applied to both the inputs and initial states of GCs, SMCs, speed cells, and direction cells (Suppl. 5.2).

After training, we randomly selected locations in the environment and sampled the corresponding ground-truth SMC responses. Each sampled response was repeated *T* times to form a query. During queries, the network state was initialized to zero across all hidden-layer neurons, and the query was input only to the spatial modulated cells, while responses from all cells were recorded over *T* timesteps. At each timestep, the activity of a subpopulation of cells (e.g., SMC, GC, HPC) was decoded into a position by finding the nearest neighbor on the corresponding subpopulation ratemap aggregated during testing (Suppl.5.4). Nearest neighbor search was performed using FAISS [60, 61] (Suppl.4).

**Figure 2:**
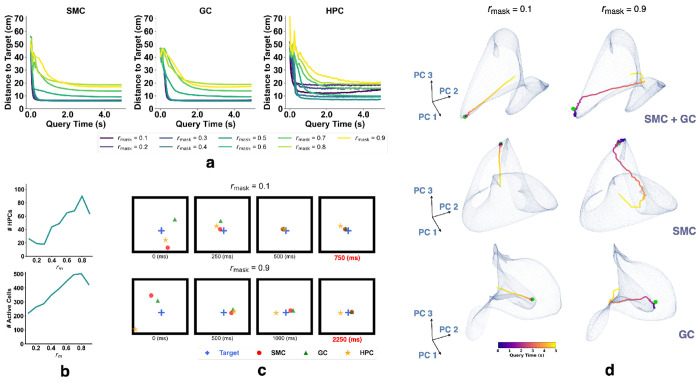
Recalling MEC representations from sensory observations with networks trained under different masking ratios *r*_mask_. **(a–d)** Results from querying the trained network with fixed sensory input. **(a)** L2 distance between decoded and target positions using SMCs, GCs, and HPCs. **(b)** Top: number of identified HPCs vs. *r*_mask_ (max 512). Bottom: number of active hippocampal units vs. *r*_mask_ (max 512). **(c)** Decoded positions from SMC, GC, and HPC population responses. **(d)** Example recall trajectories for SMC, GC, and their concatenation. Semi-transparent surfaces show PCA-reduced ratemaps (extrapolated 5× for visualization) from testing. Trajectories are colored by time; green dot marks the target.

Figure 2a shows the L2 distance between decoded and ground-truth positions over time. The network was queried for 5 seconds (100 timesteps), and all models successfully recalled the goal location with high accuracy. HPCs were identified as neurons with mean firing rates above 0.01 Hz and spatial information content (SIC) above 20 (Suppl. 2.1). The number of HPCs increased with *r*_mask_, and location decoding using HPCs was performed only for models with more than 10 HPCs. We observe a trade-off in which higher *r*_mask_ leads to more HPCs and improved decoding accuracy but reduces the network’s ability to recall sensory observations (Figure 2a,b).

To visualize the recall process (Figure 2c), we conducted Principal Components Analysis (PCA) on the recall trajectories. We first flattened the $L_{x}\times L_{y}\times N$ ground-truth ratemap into a $(L_{x}\cdot L_{y})\times N$ matrix, where *L_x_* and *L_y_* are the spatial dimensions of the arena and *N* is the number of cells in each subpopulation. PCA was then applied to reduce this matrix to $\left(L_{x}\cdot L_{y}\right)\times 3$, retaining only the first three principal components. The trajectories (colored by time), goal responses (green dot), and ratemaps collected during testing were projected into this reduced space for visualization. In Figure 2d, the recall trajectories for all subpopulations converge near the target representation, indicating successful retrieval of target SMC and GC patterns. Once near the target, the trajectories remain close and continue to circulate around it, indicating stable dynamics during the recall process.

## 4 Planning with Recalled Representations

The recall experiment above demonstrates that auto-association of spatially modulated cells (SMCs) and grid cells (GCs) through hippocampal place cells (HPCs) could enable the recall of all cells’ (GC, HPC, and SMC) representations using sensory cues. Among the recalled patterns, we propose that animals primarily use GCs for planning, as direct planning with HPCs or SMCs cannot take shortcut paths through unvisited regions. Auto-association removes the need for direct planning with SMCs or HPCs; sensory cues are required only to determine initial and target GC representations. Once recalled, a sequence of speed and direction signals drives GC activity from the initial to the target pattern, generating the planned path. Sensory patterns can then be reconstructed from intermediate GC states through HPCs, further reducing reliance on SMCs and HPCs during planning. These speed and direction signals are context-free and generalizable across environments, allowing planning strategies learned in one room to transfer to others. Finally, planning within GC representations may enable local transition rules to generalize to long-range path planning.

### 4.1 Decoding Displacement from Grid Cells

We first revisit and reframe the formalism in [44]. Grid cells are grouped into modules based on shared spatial periods and orientations. Within each module, relative phase relationships remain stable across environments [10, 58, 62, 63]. This stability allows the population response of a grid module to be represented by a single phase variable *ϕ* [44], which is a scalar in 1D and a 2D vector in 2D environments. This variable maps the population response onto an *n*-dimensional torus [64], denoted as ${𝕋}^{n}={\mathbb{R}}^{n}/2\pi {\mathbb{Z}}^{n}≅[0,2\pi {)}^{n}$, where *n* ∈ {1, 2} is the dimension of navigable space.

Consider a 1D case with *ϕ_c_* and *ϕ_t_* as the phase variables of the current and target locations in some module. The phase difference is Δ*ϕ* = *ϕ_t_* − *ϕ_c_*, and since *ϕ_c_, ϕ_t_* ∈ [0, 2*π*), we have Δ*ϕ* ∈ (−2*π*, 2*π*). However, for vector-based navigation, we instead need Δϕ* such that *ϕ_t_* = [*ϕ_c_* + Δϕ*]_2*π*_, where [·]_2*π*_ is an element-wise modulo operation so that *ϕ_t_* is defined on [0, 2*π*). Simply using Δ*ϕ* directly is not sufficient because multiple wrapped phase differences correspond to the same phase *ϕ_t_*, but different physical positions on the torus. Therefore, we restrict Δ*ϕ* to be defined on (−*π*, *π*) such that the planning mechanism always selects the shortest path on the torus that points to the target phase. The decoded displacement in physical space is then $\overset{ˆ}{d}\in [-\ell /2,\ell /2]$.

**Figure 3:**
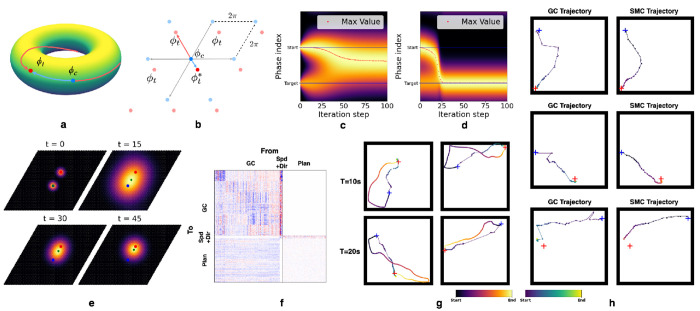
**(a)** The population response of a single grid module forms an *n*-dimensional torus, where multiple phase differences can connect the current and target phases. **(b)** Unwrapping phases into physical space yields 2^*n*^ candidate displacements; only *ϕ_t_*^∗^ lies within the principal domain (−*π*, *π*)^*n*^. **(c)** A Markovian process asymptotically identifies the most likely next phase that moves closer to the target. **(d)** Renormalizing after each update produces a smooth trajectory from start to target. **(e)** Illustration of this process in 2D space. **(f)** Learned connectivity matrix of the planning RNN using only grid cells. **(g)** Planned trajectories for targets reachable within 10 and 20 seconds. Blue and red crosses mark start and target locations; the reference line shows the full trajectory for visualization. The dots represented the decoded locations. **(h)** A planning network connected to the full HC-MEC, receiving input only from GC and controlling speed and direction, drives SMC responses to update alongside GC, tracing a trajectory closely aligned with the planned GC path.

For 2D space, we define $\Delta \phi \in {\mathbb{R}}^{2}$ on (−*π*, *π*)^2^ by treating the two non-collinear directions as independent 1D cases. In Figure 3a, the phase variables *ϕ_c_* and *ϕ_t_* correspond to two points on a 2D torus. When unwrapped into physical space, these points repeat periodically, forming an infinite lattice of candidate displacements (Figure 3b). In 2D, this yields four (2^2^) distinct relative positions differing by integer multiples of 2*π* in phase space. Only the point ϕ_t_* lies within the principal domain (−*π*, *π*)^2^, and the decoder selects Δ*ϕ* ∈ (−*π*, *π*)^2^ that minimizes ∥Δ*ϕ*∥, subject to $\phi_{t}={\left[\phi_{c}+\Delta \phi \right]}_{2\pi }$.

### 4.2 Sequential Planning

Previous network models compute displacement vectors from GCs by directly decoding from the current *ϕ_c_* and the target *ϕ_t_* [44]. However, studies show that during quiescence, GCs often fire in coordinated sequences tracing out trajectories [65, 66], rather than representing single, abrupt movements toward the target. At a minimum, long-distance displacements are not directly executable and must be broken into smaller steps. What mechanism could support such sequential planning?

We first consider a simplistic planning model on a single grid module. Phase space can be discretized into *N_ϕ_* bins, grouping GC responses into *N_ϕ_* discrete states. Local transition rules can be learned even during random exploration, allowing the animal to encode transition probabilities between neighboring locations. These transitions can be compactly represented by a matrix $T\in {\mathbb{R}}^{N_{\phi }\times N_{\phi }}$, where *T_ij_* gives the probability of transitioning from phase *i* to phase *j*. With this transition matrix, the animal can navigate to the target by stitching together local transitions, even without knowing long-range displacements. Specifically, suppose we construct a vector $v^{\text{plan}}\in {\mathbb{R}}^{N_{\phi }}$ with nonzero entries marking the current and target phases to represent a planning task. Multiplying *v*^plan^ by matrix *T* propagates the current and target phases to their neighboring phases, effectively performing a “search” over possible next steps based on known transitions.

By repeatedly applying this update, the influence of the current and target phase spreads through phase space, eventually settling on an intermediate phase that connects start and target (Figure 3c). If the animal selects the phase with the highest value after each update and renormalizes the vector, this process traces a smooth trajectory toward the target (Figure 3d). This approach can be generalized to 2D phase spaces (Figure 3e). In essence, we propose that the animal can decompose long-range planning into a sequence of local steps by encoding a transition probability over phases in a matrix. A readout mechanism can then map these phase transitions into corresponding speeds and directions and subsequently update GC activity toward the target. We also note that this iterative planning process resembles search-based methods in robotics, such as RRT-Connect [67].

### 4.3 Combining Decoded Displacement from Multiple Scales

Our discussions of planning and decoding in sections 4.1 and 4.2 were limited to displacements within a single grid module. However, this is insufficient when Δ*ϕ* exceeds half the module’s spatial period (*ℓ*/2). The authors of [44] proposed combining Δ*ϕ* across modules before decoding displacement. We instead suggest decoding Δ*ϕ* within each module first, then averaging the decoded displacements across modules is sufficient for planning. This procedure allows each module to update its phase using only local transition rules while still enabling the animal to plan a complete path to the target.

We start again with the 1D case. Suppose there are *m* different scales of grid cells, with each scale *i* having a spatial period *ℓ_i_*. From the smallest to the largest scale, these spatial periods are *ℓ*_1_, …, *ℓ_m_*. The grid modules follow a fixed scaling factor *s*, which has a theoretically optimal value of *s* = *e* in 1D rooms and $s=\sqrt{e}$ in 2D [12]. Thus, the spatial periods satisfy *ℓ_i_* = *ℓ*_0_ · *s^i^* for *i* = 1, …, *m*, where *ℓ*_0_ is a constant parameter that does not correspond to an actual grid module.

Given the ongoing debate about whether grid cells contribute to long-range planning [68, 69], we focus on mid-range planning, where distances are of the same order of magnitude as the environment size. Suppose two locations in 1D space are separated by a ground truth displacement $d\in {\mathbb{R}}_{+}$, bounded by half the largest scale (*ℓ_m_*/2). We can always find an index *k* where *ℓ*_1_/2, …, *ℓ_k_*/2 ≤ *d* < *l*_*k*+1_/2 < … , *ℓ_m_*/2. Given *k*, we call scales *ℓ*_*k*+1_, …, *ℓ_m_* **decodable** and scales *ℓ*_1_, …, *ℓ_k_* **undercovered**. For undercovered scales, phase differences Δ*ϕ* are wrapped around the torus at least one period of (−*π*, *π*) and may point to the wrong direction. We thus denote phase difference from undercovered scales as *Z_i_*. If we predict displacement by simply averaging the decoded displacements from all grid scales, the predicted displacement is:

$$\overset{ˆ}{d}=\frac{\ell_{0}}{2\pi m}\cdot \left(\sum_{i=1}^{k}s^{i}\cdot Z_{i}+\sum_{i=k+1}^{m}s^{i}\cdot \Delta \phi_{i}\right)$$

In 1D, the remaining distance after taking the predicted displacement is $d_{\text{next}}=d_{\text{current}}-\overset{ˆ}{d}$ For the predicted displacement to always move the animal closer to the target, meaning $d_{\text{next}}<d_{\text{current}}$, it suffices that $m>k+\frac{1-s^{-k}}{s-1}$ (see Suppl. 1.1). This condition is trivially satisfied in 1D for s=e, as $\frac{1-s^{-k}}{s-1}<1$ requiring only *m* > *k*. In 2D, where the optimal scaling factor $s=\sqrt{e}$, the condition tightens slightly to *m* > *k* + 1. Importantly, as the animal moves closer to the target, more scales become decodable, enabling increasingly accurate predictions that eventually lead to the target. In 2D, planning can be decomposed into two independent processes along non-collinear directions. Although prediction errors in 2D may lead to a suboptimal path, this deviation can be reduced by increasing the number of scales or taking smaller steps along the decoded direction, allowing the animal to gradually approach the target with higher prediction accuracy.

### 4.4 A RNN Model of Planning

#### Planning Using Grid Cells Only.

We test our ideas in an RNN framework. We first ask whether a planner subnetwork, modeled together with a GC subnetwork, can generate sequential trajectories toward the target using only grid cell representations. Accordingly, we connect a planning network to a pre-trained GC network that has already learned path integration. For each planning task, the GC subnetwork is initialized with the ground truth GC response at the start location, while the planner updates the GC state from the start to the target location’s GC response by producing a sequence of feasible actions—specifically, speeds and directions. This ensures the planner generates feasible actions rather than directly imposing the target state on the GCs, while a projection from the GC region to the planning region keeps the planner informed of the current GC state. The planner additionally receives the ground truth GC response of the target location through a learnable linear projection. At each step, the planner receives $W^{in}g^{*}+W^{g\to p}g_{t}\in {\mathbb{R}}^{d_{p}}$, where g* and *g_t_* are the goal and current GC patterns, while $W^{in}$ and $W^{g\to p}$ are the input and GC-to-planner projection matrices. This combined input has the same dimension as the planner network. Conceptually, it can be interpreted as the planning vector *v*^plan^, while the planner’s recurrent matrix represents the transition matrix *T*. The resulting connectivity matrix is shown in Figure 3f.

During training, we generate random 1-second trajectories to sample pairs of current and target locations, allowing the animal to learn local transition rules. These trajectories are used only to ensure that the target location is reachable within 1 second from the current location; the trajectories themselves are not provided to the planning subnetwork. The planning subnetwork is trained to minimize the mean squared error between the current and target GC states for all timesteps.

For testing, we generate longer 10 and 20 second trajectories to define start and target locations, again without providing the full trajectories to the planner. The GC states produced during planning are decoded at each step to infer the locations the animal is virtually planned to reach. As shown in Figure 3g, the dots represent these decoded locations along the planned path, while the colored line shows the full generated trajectory for visualization and comparison. We observe that the planner generalizes from local to long-range planning and can take paths that shortcut the trajectories used to generate start and target locations. Notably, even when trained on just 128 fixed start-end pairs over 100 steps, it still successfully plans paths between locations reachable over 10 seconds.

#### Planning with HC-MEC Enables Reconstruction of Sensory Experiences Along Planned Paths.

We next test whether the HC-MEC loop enables the network to reconstruct sensory experiences along planned trajectories using intermediate GC states. To this end, we connect an untrained planning subnetwork to a pre-trained HC-MEC model (*r*_mask_ = 1.0, see Suppl. 5.2) and fix all projections from SMCs and HPCs to the planner to zero. This ensures the planner uses only GC representations for planning and controls HC-MEC dynamics by producing inputs to speed and direction cells. SMCs and GCs are initialized to their respective ground truth responses at the start location.

Using the same testing procedures as before, we sampled the SMC and GC responses while the planner generated paths between two locations reachable within 10 seconds. We decoded GC and SMC activity at each timestep to locations using nearest neighbor search on their respective ratemaps. We found that the decoded SMC trajectories closely followed those of the GCs, suggesting that SMC responses can be reconstructed from intermediate GC states via HPCs (see Figure 3h). Additionally, compared to the GC-only planning case, we reduced the number of GCs in the HC-MEC model to avoid an overly large network, which would make the HC-MEC training hard to converge. Although this resulted in a less smooth GC-decoded trajectory than in Figure 3g, the trajectory decoded from SMCs was noticeably smoother. We suggest this is due to the auto-associative role of HPCs, which use relayed GC responses to reconstruct SMCs, effectively smoothing the trajectory.

## 5 Discussion

Decades of theoretical and computational research have sought to explain how and why hippocampal place cells (HPCs) and grid cells (GCs) emerge. These models address the question “What do they encode?” But an equally important question is “Why does the brain encode?” One answer is that animals develop and maintain place and grid representations to support intrinsically motivated navigation, enabling access to resources critical to survival, such as food, water, and shelter. Thus, we take the perspective: *given the known phenomenology of GCs and HPCs, how might they be wired together to support intrinsically motivated navigation?*

Previous studies show that HPCs are linked to memory and context but have localized spatial representations lacking the relational structure needed for planning. In contrast, the periodic lattice of GCs supports path planning by generalizing across environments, but weak contextual tuning limits direct recall of GC patterns from sensory cues. Rather than viewing GCs and HPCs as parallel spatial representations, we propose that GCs and spatially modulated cells (SMCs), reflecting sensory observations, form parallel, independent localization systems, each encoding different aspects of space. HPCs then link these representations through auto-association, enabling recovery of one when the other fails and providing a higher-level, abstract spatial representation.

Testing these ideas, we built a single-layer RNN that simultaneously models GCs, HPCs, SMCs, and a planning subnetwork. We showed that auto-association in HPCs, linking MEC sensory (SMCs) and spatial (GCs) representations enables recall of GC patterns from contextual cues. With recalled GC patterns, local planning using only GCs can be learned and generalized to longer trajectories. Finally, HPCs can reconstruct sensory experiences along planned paths, obviating the need for direct planning based solely on HPCs or SMCs. Our model is flexible and accommodates existing theories, as follows. Recurrent connections from MEC cause HPC activity to lag one timestep behind MEC inputs; during pattern completion, HPCs implicitly learn to predict the next timestep’s MEC inputs, and thus can account for the successor representation theory of HPCs [36]. This predictive nature is thus consistent with previous theories that predictive coding in HPCs could support planning [37], but we emphasize that using GCs may be more efficient when it is necessary to shortcut unvisited locations. Lastly, while we propose that the HC–MEC coupling enables planning through recall and reconstruction, a parallel idea was proposed in [70], where the same coupling was argued to support increased episodic memory capacity.

Our theory makes several testable predictions. First, sensory cues should reactivate GC activity associated with a target location, even when the animal is stationary or in a different environment. Second, inhibiting MEC activity should impair path planning and disrupt sequential preplay in the HC. Finally, if hippocampal place cells reconstruct sensory experiences during planning, disrupting MEC-to-HC projections should impair goal-directed navigation, while disrupting HC-to-MEC feedback should reduce planning accuracy by preventing animals from validating planned trajectories internally.

### Limitations:

First, existing emergence models of GCs make it difficult to precisely control GC scales and orientations [11, 26], and so to avoid the complicating analysis of the simultaneous emergence of GCs and HPCs, we supervised GC responses during training. Future work on their co-emergence could further support our proposed planning mechanism. Second, our framework does not account for boundary avoidance, which would require extending the HC-MEC model to include boundary cell representations [40, 71, 72]. Finally, our discussion of planning with GCs assumes the environment is of similar scale to the largest grid scale. One possibility is long-range planning may involve other brain regions [69], as suggested by observations that bats exhibit only local 3D grid lattices without a global structure [68]. Animals might use GCs for mid-range navigation, while global planning stitches together local displacement maps from GC activity.

## Supplementary Material

1
