# REMI: Reconstructing Episodic Memory During Internally Driven Path Planning

**DOI:** 10.1101/2025.07.02.662824

**Published:** 2025-10-24

**Authors:** Zhaoze Wang, Genela Morris, Dori Derdikman, Pratik Chaudhari, Vijay Balasubramanian

**Affiliations:** 1 Dept. of Electrical and Systems Eng., Univ. of Pennsylvania; 2 Tel Aviv Sourasky Medical Center; 3 Gray Faculty of Medical and Health Sciences, Tel Aviv University; 4 Rappaport Faculty of Medicine, Technion – Israel Institute of Technology; 5 Dept. of Physics, Univ. of Pennsylvania; 6 Santa Fe Institute; 7 Rudolf Peierls Centre for Theoretical Physics, University of Oxford

## Abstract

Grid cells in the medial entorhinal cortex (MEC) and place cells in the hippocampus (HC) both form spatial representations. Grid cells fire in triangular grid patterns, while place cells fire at specific locations and respond to contextual cues. How do these interacting systems support not only spatial encoding but also internally driven path planning, such as navigating to locations recalled from cues? Here, we propose a system-level theory of MEC-HC wiring that explains how grid and place cell patterns could be connected to enable cue-triggered goal retrieval, path planning, and reconstruction of sensory experience along planned routes. We suggest that place cells autoassociate sensory inputs with grid cell patterns, allowing sensory cues to trigger recall of goal-location grid patterns. We show analytically that grid-based planning permits shortcuts through unvisited locations and generalizes local transitions to long-range paths. During planning, intermediate grid states trigger place cell pattern completion, reconstructing sensory experiences along the route. Using a single-layer RNN modeling the HC-MEC loop with a planning subnetwork, we demonstrate these effects in both biologically grounded navigation simulations using RatatouGym and visually realistic navigation tasks using Habitat Sim. Codes for experiments, simulations, and vision encoder are available at ^1,2,3^.

## 1 Introduction

Gridandplacecellsinthemedialentorhinalcortex(MEC)andhippocampus(HC)formcomplementary spatial codes for navigation [1–3]. Grid cells (GCs) in MEC fire in periodic triangular patterns as animals move through space [4–6], spanning multiple spatial scales [7–10] potentially shaped by inhibitory gradients within attractor networks [11]. Path integration theories propose that GCs track position by integrating movement [4, 12, 13], and recurrent neural networks (RNNs) trained for this task similarly develop grid-like activity [14–18]. Hippocampal place cells (HPCs) fire at specific locations [1, 2] and remap across contexts [19–23], forming sparse, orthogonal representations that minimize contextual interference [24–26]. They auto-associate sensory sequences relayed through MEC during exploration [27–30], linking related memories via continuous attractor dynamics [31–34], and emerge in RNNs that auto-encode spatial experience [21–23].

Despite extensive study of grid and place cell function [14, 16, 18, 21–23, 35, 36], it remains unclear how these representations support internally driven navigation, such as recalling goals from partial cues and planning novel routes. This ability is fundamental to flexible, goal-directed behavior, allowing animals to adapt to novel situations without extensive relearning. Evidence from rodent rest and sleep shows that hippocampal sequences replay past trajectories and generate novel ones through unvisited locations [37, 38], suggesting an underlying mechanism for offline planning.

Two complementary frameworks have been proposed to explain planning. The successor representation (SR) posits that hippocampal place cells encode expected future occupancy [39, 40], enabling flexible replanning when goals change [41, 42] and supporting generalization across related tasks through reusable predictive representations [43]. However, SR models discretize space into learned transition states, requiring experience at many locations and limiting interpolation to unvisited areas. Grid-cell models instead propose continuous, periodic codes that support vector-based navigation [44], with stable phase relationships providing context-independent spatial metrics. Yet encoding pairwise relations among locations becomes intractable as environments scale, and pure grid-based planning cannot easily capture context-specific constraints or hippocampal replay phenomena. Each framework offers a partial explanation of the problem, but how the brain integrates context-dependent memory with context-independent spatial metrics remains unresolved.

Here, we propose a unified theory of hippocampal-entorhinal circuitry that explains how internally driven navigation arises from the interaction between place and grid cells. This specific hippocampal-entorhinal wiring can enable cue-triggered goal retrieval and grid-based planning. Building on recent work showing that place cells autoassociatively encode sensory inputs [22], we propose that they also associate these inputs with GC activity. This coupling supports bidirectional pattern completion, whereby sensory cues can reactivate corresponding grid representations to guide planning, and grid states can reconstruct the expected sensory experiences along planned routes.

We verified these effects using a single RNN trained to autoencode sensory inputs, integrate velocity signals for path integration, and form autoassociative links between sensory and grid representations. Through training, the RNN developed an internal representation of the navigable space. To enable planning, we expanded the RNN’s hidden layer to include an additional planner subnetwork that drives the encoder’s dynamics with internally generated action sequences, enabling the system to traverse imagined paths between the current location and a recalled goal. To test whether this framework could generalize to realistic navigation, we replaced simulated sensory patterns with visual features from panoramic images in a photorealistic environment (Habitat-Sim [45–47]), encoded using a modified Masked Autoencoder (MAE) [48]. During planning, as the network traversed imagined trajectories, intermediate sensory states were decoded by the MAE into images that closely matched the expected views at corresponding locations.

Our theory predicts that (a) during planning, HC-MEC coupling will induce “look forward” sweeps in MEC spatial representation, resembling recent experimental results [49], (b) when presented with sensory cues associated with distant locations, grid cell response patterns corresponding to those locations will be reactivated, and (c) disrupting MEC-to-HC projections should impair goal-directed navigation, while disrupting HC-to-MEC feedback should reduce planning accuracy.

## 2 Method

### Conceptual Framework of the HC-MEC Loop.

We construct a conceptual framework showing how known spatial cell types could integrate into a unified system supporting both mapping and internally driven planning. Our theory builds on four established theories: **(a)** GCs arise from integrating velocity sequences for localization [4, 12–18]; **(b)** spatially modulated cells (SMCs) in MEC respond to external sensory stimuli [27] and relay sensory inputs to hippocampus; **(c)** HPCs autoassociate noisy sensory inputs from SMCs to reconstruct denoised sensory experience during recall [21, 22, 50–52]; and **(d)** while HPCs may also support planning, GCs provide a more efficient substrate for planning through their continuous, context-independent encoding [44].

Since both GCs and SMCs are present in MEC and both project to hippocampus, GCs may be regarded as a special form of sensory response derived from proprioceptive signals. This anatomical arrangement suggests our core theoretical contribution, extending (c) by proposing HPCs autoassociate and pattern-complete not only SMCs but also GCs. When sensory cues are unreliable and path integration drifts over time due to noise, this association provides a natural solution for more robust localization than either system alone. This framework directly yields three key predictions: (1) partial sensory cues can reactivate the corresponding grid cell state via hippocampal autoassociation; (2) the recalled grid state can guide planning on the grid cell manifold, enabling efficient, context-independent, and generalizable path computation; (3) during planning, intermediate grid states trigger hippocampal reactivation of the corresponding sensory representations, reconstructing the expected sensory experience along the planned route.

### An RNN Model of the HC-MEC Loop.

Our theoretical framework requires an RNN that can simultaneously (1) autoencode masked and noisy sensory experiences (SMCs), (2) contain grid cells capable of path-integrating noisy movement inputs, (3) form autoassociative links between SMC and GC responses, and (4) later support planning. To achieve these, we extend RNN formulations from previous models of spatial navigation cell types [14, 16, 18, 22, 53]. Specifically, consider a standard RNN that updates its dynamics as:

(1) $$z_{t+1}=\alpha \cdot z_{t}+(1-\alpha )\cdot \left(W^{in}u_{t}+W^{rec}f\left(z_{t}\right)\right)$$

where $z\in R^{d_{z}}$ is the hidden state, u is the input, α is the forgetting rate, and $W^{\text{in}},\;W^{\text{rec}}$ are the input and recurrent weight matrices. The output is given by a linear readout: $y_{t}=W^{\text{out}}z_{t}\in R^{d_{o}}$. We extend this RNN by introducing auxiliary input and output nodes, $z^{I}$ and $z^{O}$, and update as:

(2) $$\left[\begin{array}{c} z_{t+1}\\ z_{t+1}^{I}\\ z_{t+1}^{O} \end{array}\right]=\alpha ⊙\left[\begin{array}{c} z_{t}\\ z_{t}^{I}\\ z_{t}^{O} \end{array}\right]+(1-\alpha )⊙\left(\left[\begin{array}{c} 0\\ u_{t}\\ 0 \end{array}\right]+\left[\begin{array}{ccc} W^{\mathrm{rec}} & {\tilde{W}}^{\mathrm{in}} & W^{\left(13\right)}\\ W^{\left(21\right)} & W^{\left(22\right)} & W^{\left(23\right)}\\ {\tilde{W}}^{\mathrm{out}} & W^{\left(32\right)} & W^{\left(33\right)} \end{array}\right]f\left(\left[\begin{array}{c} z_{t}\\ z_{t}^{I}\\ z_{t}^{O} \end{array}\right]\right)\right)$$

Here, $z^{I}$ directly integrates the input $u_{t}$ without a learnable projection, while $z^{O}$ is probed and supervised to match simulated ground-truth cell responses. This design eliminates the need for projection matrices $W^{\mathrm{in}}$ and $W^{\mathrm{out}}$. Instead, ${\tilde{W}}^{\mathrm{in}}$ and ${\tilde{W}}^{\mathrm{out}}$ act as surrogate mappings for the original projections. We set α as a learnable vector in $R^{d_{z}+d_{I}+d_{O}}$ to allow different cells to have distinct forgetting rates, with ⊙ denoting element-wise multiplication.

### Supervising Spatially Modulated Cells (SMCs).

The SMCs are set as both input and output nodes, trained to reproduce the simulated ground truth. At each time step, SMC units receive simulated but partially occluded sensory inputs representing noisy environmental observations. After each recurrent update, the network is expected to reconstruct the corresponding noiseless sensory pattern and is penalized for deviations of the SMC subpopulation from the ground truth responses. These SMCs are assumed to primarily respond to sensory cues during physical traversal. Supervision constrains their dynamics to reflect tuning to these signals, while learned recurrent connections formed during training are intended to reflect Hebb-like updates in the brain that preserve this tuning structure.

### Supervising Grid Cells for Path-Integration.

Our theoretical model also requires grid cells that have learned to perform path integration. To achieve this, we assign two additional subpopulations within the RNN’s hidden units to represent speed and allocentric head direction cells. Both subpopulations are defined as input nodes that only receive external inputs. Head direction cells are assigned preferred allocentric directions uniformly distributed over [0,2π) with a fixed angular tuning width, ensuring non-negative responses and a population activity that lies on a 1D ring (see Suppl. 3). We also simulate ground-truth grid cell activity maps. To train the network to perform path integration, the model is provided only with the initial grid cell responses at t=0. For all subsequent timesteps, the network must infer the grid cell activity from the initial response together with the ongoing inputs from the speed and head direction cells.

**Figure 1:**
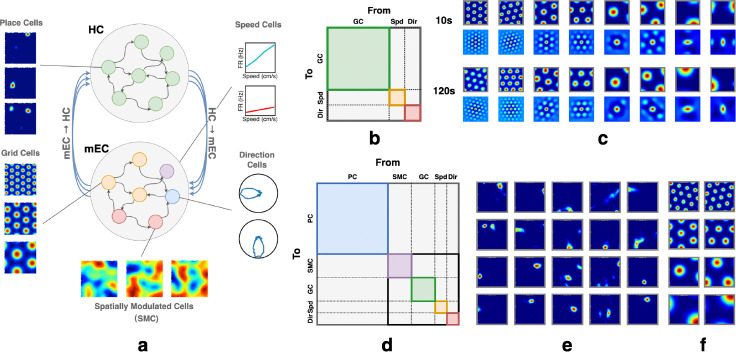
**(a)** RNN model of the HC-MEC loop. The top subnetwork contains HPCs, with example emergent place fields shown on the left. The bottom subnetwork includes partially supervised GCs, as well as supervised speed, head direction, and spatially modulated cells (SMCs); example grid fields shown at left. **(b)** & **(d)** Speed cells and head direction cells are denoted as *Spd* and *Dir*, respectively. Colored regions highlight within-group recurrent connectivity to indicate the partitioning of the connectivity matrix by cell groups. However, at initialization, no structural constraints are enforced. The full connectivity matrix is randomly initialized. **(b)** Illustration of the path-integration network’s connectivity matrix. **(c)** The network is trained to path-integrate 5s trials and tested on 10s trials (L=100.50±8.49cm); the grid fields remain stable even in trials up to 120s (L=1207.89±30.98cm). For each subpanel (10s, 120s): top row shows firing fields; bottom row shows corresponding autocorrelograms. **(d)** Illustration of RNN connectivity matrix of full HC-MEC loop. **(e-f)** Example place fields (emergent) and grid fields in the full HC-MEC RNN model.

This path integration component can be viewed as a modified version of the networks described in [14–16, 18]. To verify that the network indeed learned path integration, we first trained a model containing only grid, speed, and head direction cells (Figure 1b). We simulated six grid modules with spatial periods scaled by the theoretical optimal factor $\sqrt{e}$ [54]. The smallest grid spacing is set to 30 cm, defined as the distance between two firing centers of a grid cell [55]. The grid spacing to field size ratio is 3.26 [56], with firing fields modeled as Gaussian blobs with radii equal to two standard deviations. We train this model on short random trajectories (5 s) but accurately path-integrate over significantly longer trajectories (10 s, 120 s) during testing (Figure 1c).

### Emergence of Place Cell-Like Patterns During Autoassociation of SMCs and GCs.

The central idea of our HC-MEC model is that localization using sensory inputs (SMCs) or through path integration alone can each fail under noise. Associating the two representations through hippocampal place cells makes localization more robust. To model this, we introduced a subpopulation of hidden units in the RNN that are neither input nor output neurons. They do not directly receive external signals or produce outputs. These units receive input from and project to both the SMC and GC subpopulations through recurrent connections (Figure 1d). After training the full HC-MEC network, with SMC units supervised to autoencode masked sensory inputs and GC units trained to path integrate noisy movement signals, we observed the spontaneous emergence of place cell-like activity in this intermediary region. No explicit supervision or spatial constraint was imposed, and the place-like patterns emerged naturally through training, consistent with previous findings [22].

## 3 Recalling MEC Representations from Sensory Observations

We test our first prediction that associating sensory observations (SMC responses) with GC patterns enables recall of those patterns from partial sensory cues. Auto-association arises when input patterns are incomplete or degraded. To model this, we trained nine identical networks differing only in masking ratio $r_{\text{mask}}$ (0.1 to 0.9), which specifies the maximum fraction of head direction, speed, GC, and SMC inputs and initial hidden states randomly set to zero during training. Each model was trained on randomly sampled short trajectories using a fixed $r_{\text{mask}}$, with new masks generated for every trajectory and varying across time and cells. Masks were applied to both inputs and initial states of GCs, SMCs, speed cells, and head direction cells (Suppl. 5.2).

After training, we randomly selected locations in the environment and sampled the corresponding ground-truth SMC responses. Each sampled response was repeated T times to form a query. During queries, the network state was initialized to zero across all hidden-layer neurons, and the query was input only to the spatial modulated cells, while responses from all cells were recorded over T timesteps. At each timestep, the activity of a subpopulation of cells (e.g., SMC, GC, HPC) was decoded into a position by finding the nearest neighbor on the corresponding subpopulation ratemap aggregated during testing (Suppl.5.4). Nearest neighbor search was performed using FAISS [57, 58] (Suppl.4).

**Figure 2:**
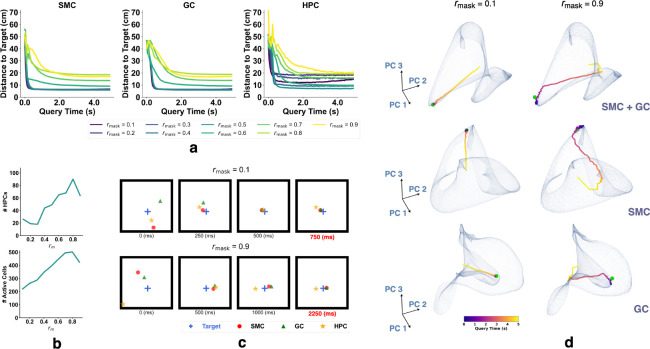
Recalling MEC representations from sensory observations with networks trained under different masking ratios $r_{\text{mask}}$. **(a–d)** Results from querying the trained network with fixed sensory input. **(a)** L2 distance between decoded and target positions using SMCs, GCs, and HPCs. **(b)** Top: number of identified HPCs vs. $r_{\text{mask}}$ (max 512). Bottom: number of active hippocampal units vs. $r_{\text{mask}}$ (max 512). **(c)** Decoded positions from SMC, GC, and HPC population responses. **(d)** Example recall trajectories for SMC, GC, and their concatenation. Semi-transparent surfaces show PCA-reduced ratemaps (extrapolated 5× for visualization) from testing. Trajectories are colored by time; green dot marks the target.

Figure 2a shows the L2 distance between decoded and ground-truth positions over time. The network was queried for 5 seconds (100 timesteps), and all models successfully recalled the goal location with high accuracy. HPCs were identified as neurons with mean firing rates above 0.01 Hz and spatial information content (SIC) above 20 (Suppl. 2). The number of HPCs increased with $r_{\text{mask}}$, and location decoding using HPCs was performed only for models with more than 10 HPCs. We observe a trade-off in which higher $r_{\text{mask}}$ leads to more HPCs and improved decoding accuracy but reduces the network’s ability to recall sensory observations (Figure 2a,b).

To visualize the recall process (Figure 2c), we conducted Principal Components Analysis (PCA) on the recall trajectories. We first flattened the $L_{x}\times L_{y}\times N$ ground-truth ratemap into a $\left(L_{x}\cdot L_{y}\right)\times N$ matrix, where $L_{x}$ and $L_{y}$ are the spatial dimensions of the arena and N is the number of cells in each subpopulation. PCA was then applied to reduce this matrix to $\left(L_{x}\cdot L_{y}\right)\times 3$, retaining only the first three principal components. The trajectories (colored by time), goal responses (green dot), and ratemaps collected during testing were projected into this reduced space for visualization. In Figure 2d, the recall trajectories for all subpopulations converge near the target representation, indicating successful retrieval of target SMC and GC patterns. Once near the target, the trajectories remain close and continue to circulate around it, indicating stable dynamics during the recall process.

## 4 Planning with Recalled Representations

The recall experiment shows that auto-associating spatially modulated cells (SMCs) and grid cells (GCs) through hippocampal place cells (HPCs) enables recovery of all cells’ representations from partial sensory cues. Although planning with HPCs and SMCs is feasible [40–43], their context dependence limits generalization across environments. In contrast, GC patterns encode the geometric structure of space and provide a context-independent substrate for planning. Planning on the grid manifold allows HPC auto-association to reconstruct corresponding SMC representations from intermediate GC states, removing the need to plan directly with HPCs or SMCs. We therefore propose that planning occurs on the grid cell manifold, with sensory details later recovered through hippocampus auto-associations.

### 4.1 Decoding Displacement from Grid Cells

We first revisit and reframe the formalism in [44]. Grid cells are grouped into modules based on shared spatial periods and orientations. Within each module, relative phase relationships remain stable across environments [10, 55, 59, 60]. This stability allows the population response of a grid module to be represented by a single phase variable ϕ [44], which is a scalar in 1D and a 2D vector in 2D environments. This variable maps the population response onto an n-dimensional torus [61], denoted as $T^{n}=R^{n}/2\pi Z^{n}≅[0,\;2\pi {)}^{n}$, where n∈{1,2} is the dimension of navigable space.

Consider a 1D case with $\phi_{c}$ and $\phi_{t}$ as the phase variables of the current and target locations in some module. The phase difference is $\Delta \phi =\phi_{t}-\phi_{c}$, and since $\phi_{c},\phi_{t}\in [0,\;2\pi )$, we have Δϕ∈(−2π,2π). However, for vector-based navigation, we instead need $\Delta \phi^{*}$ such that $\phi_{t}={\left[\phi_{c}+\Delta \phi^{*}\right]}_{2\pi }$, where $[\cdot {]}_{2\pi }$ is an element-wise modulo operation so that $\phi_{t}$ is defined on [0,2π). Simply using ∆ϕ directly is not sufficient because multiple wrapped phase differences correspond to the same phase $\phi_{t}$, but different physical positions on the torus. Therefore, we restrict ∆ϕ to be defined on (−π,π) such that the planning mechanism always selects the shortest path on the torus that points to the target phase. The decoded displacement in physical space is then $\overset{ˆ}{d}\in [-\ell /2,\ell /2]$.

**Figure 3:**
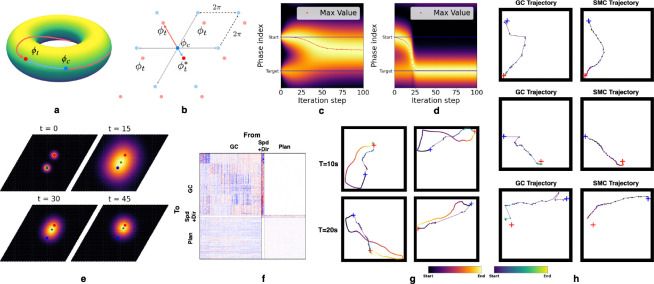
**(a)** The population response of a single grid module forms an n-dimensional torus, where multiple phase differences can connect the current and target phases. **(b)** Unwrapping phases into physical space yields $2^{n}$ candidate displacements; only $\phi_{t}^{*}$ lies within the principal domain $(-\pi ,\pi {)}^{n}$. **(c)** A Markovian process asymptotically identifies the most likely next phase that moves closer to the target. **(d)** Renormalizing after each update produces a smooth trajectory from start to target. **(e)** Illustration of this process in 2D space. **(f)** Learned connectivity matrix of the planning RNN using only grid cells. **(g)** Planned trajectories for targets reachable within 10 and 20 seconds. Blue and red crosses mark start and target locations; the reference line shows the full trajectory for visualization. The dots represented the decoded locations. **(h)** A planning network connected to the full HC-MEC, receiving input only from GC and controlling speed and direction, drives SMC responses to update alongside GC, tracing a trajectory closely aligned with the planned GC path.

For 2D space, we define $\Delta \phi \in R^{2}$ on ${\left(-\pi ,\pi \right)}^{2}$ by treating the two non-collinear directions as independent 1D cases. In Figure 3a, the phase variables $\phi_{c}$ and $\phi_{t}$ correspond to two points on a 2D torus. When unwrapped into physical space, these points repeat periodically, forming an infinite lattice of candidate displacements (Figure 3b). In 2D, this yields four (2^2^) distinct relative positions differing by integer multiples of 2π in phase space. Only the point $\phi_{t}^{*}$ lies within the principal domain ${\left(-\pi ,\pi \right)}^{2}$, and the decoder selects $\Delta \phi \in {\left(-\pi ,\pi \right)}^{2}$ that minimizes ‖Δϕ‖, subject to $\phi_{t}={\left[\phi_{c}+\Delta \phi \right]}_{2\pi }$.

### 4.2 Sequential Planning

Previous network models compute displacement vectors from GCs by directly decoding from the current $\phi_{c}$ and the target $\phi_{t}$ [44]. However, studies show that during quiescence, GCs often fire in coordinated sequences tracing out trajectories [62, 63], rather than representing single, abrupt movements toward the target. At a minimum, long-distance displacements are not directly executable and must be broken into smaller steps. What mechanism could support such sequential planning?

We first consider a simplistic planning model on a single grid module. Phase space can be discretized into $N_{\phi }$ bins, grouping GC responses into $N_{\phi }$ discrete states. Local transition rules can be learned even during random exploration, allowing the animal to encode transition probabilities between neighboring locations. These transitions can be compactly represented by a matrix $T\in R^{N_{\phi }\times N_{\phi }}$, where $T_{ij}$ gives the probability of transitioning from phase i to phase j. With this transition matrix, the animal can navigate to the target by stitching together local transitions, even without knowing long-range displacements. Specifically, suppose we construct a vector $v^{\text{plan}}\in R^{N_{\phi }}$ with nonzero entries marking the current and target phases to represent a planning task. Multiplying $v^{\text{plan}}$ by matrix T propagates the current and target phases to their neighboring phases, effectively performing a “search” over possible next steps based on known transitions.

By repeatedly applying this update, the influence of the current and target phase spreads through phase space, eventually settling on an intermediate phase that connects start and target (Figure 3c). If the animal selects the phase with the highest value after each update and renormalizes the vector, this process traces a smooth trajectory toward the target (Figure 3d). This approach can be generalized to 2D phase spaces (Figure 3e). In essence, we propose that the animal can decompose long-range planning into a sequence of local steps by encoding a transition probability over phases in a matrix. A readout mechanism can then map these phase transitions into corresponding speeds and directions and subsequently update GC activity toward the target.

### 4.3 Combining Decoded Displacement from Multiple Scales

Our discussions of planning and decoding in sections 4.1 and 4.2 were limited to displacements within a single grid module. However, this is insufficient when ∆ϕ exceeds half the module’s spatial period (ℓ/2). The authors of [44] proposed combining ∆ϕ across modules before decoding displacement. We instead suggest decoding ∆ϕ within each module first, then averaging the decoded displacements across modules is sufficient for planning. This procedure allows each module to update its phase using only local transition rules while still enabling the animal to plan a complete path to the target.

We start again with the 1D case. Suppose there are m different scales of grid cells, with each scale i having a spatial period $\ell_{i}$. From the smallest to the largest scale, these spatial periods are $\ell_{1},\ldots ,\ell_{m}$. The grid modules follow a fixed scaling factor s, which has a theoretically optimal value of s=e in 1D rooms and $s=\sqrt{e}$ in 2D [54]. Thus, the spatial periods satisfy $\ell_{i}=\ell_{0}\cdot s^{i}$ for i=1,…,m, where $\ell_{0}$ is a constant parameter that does not correspond to an actual grid module.

Given the ongoing debate about whether grid cells contribute to long-range planning [64, 65], we focus on mid-range planning, where distances are of the same order of magnitude as the environment size. Suppose two locations in 1D space are separated by a ground truth displacement $d\in R_{+}$, bounded by half the largest scale ($\ell_{m}/2$). We can always find an index k where $\ell_{1}/2,\ldots ,\ell_{k}/2\leq d<\ell_{k+1}/2<\ldots ,\ell_{m}/2$. Given k, we call scales $\ell_{k+1},\ldots ,\ell_{m}$ **decodable** and scales $\ell_{1},\ldots ,\ell_{k}$ **undercovered**. For undercovered scales, phase differences ∆ϕ are wrapped around the torus at least one period of (−π,π) and may point to the wrong direction. We thus denote the phase difference due to the undercovered scales by $Z_{i}$. If we predict displacement by simply averaging the decoded displacements from all grid scales, the predicted displacement

$$\overset{ˆ}{d}=\frac{\ell_{0}}{2\pi m}\cdot \left(\sum_{i=1}^{k}\;\;s^{i}\cdot Z_{i}+\sum_{i=k+1}^{m}\;\;s^{i}\cdot \Delta \phi_{i}\right).$$

In 1D, the remaining distance after taking the predicted displacement is $d_{\text{next}}=d_{\text{current}}-\overset{ˆ}{d}$. For the predicted displacement to always move the animal closer to the target, meaning $d_{\text{next}}<d_{\text{current}}$, it suffices that $m>k+\frac{1-s^{-k}}{s-1}$ (see Suppl. 1). This condition is trivially satisfied in 1D for s=e, as $\frac{1-s^{-k}}{s-1}<1$ requiring only m>k. In 2D, where the optimal scaling factor $s=\sqrt{e}$, the condition tightens slightly to m>k+1. Importantly, as the animal moves closer to the target, more scales become decodable, enabling increasingly accurate predictions that eventually lead to the target. In 2D, planning can be decomposed into two independent processes along non-collinear directions. Although prediction errors in 2D may lead to a suboptimal path, this deviation can be reduced by increasing the number of scales or taking smaller steps along the decoded direction, allowing the animal to gradually approach the target with higher prediction accuracy.

### 4.4 A RNN Model of Planning

#### Planning Using Grid Cells Only.

We test our ideas in an RNN framework. We first ask whether a planner subnetwork, modeled together with a GC subnetwork, can generate sequential trajectories toward the target using only grid cell representations. Accordingly, we connect a planning network to a pre-trained GC network that has already learned path integration. For each planning task, the GC subnetwork is initialized with the ground truth GC response at the start location, while the planner updates the GC state from the start to the target location’s GC response by producing a sequence of feasible actions—specifically, speeds and directions. This ensures the planner generates feasible actions rather than directly imposing the target state on the GCs, while a projection from the GC region to the planning region keeps the planner informed of the current GC state. The planner additionally receives the ground truth GC response of the target location through a learnable linear projection. At each step, the planner receives $W^{in}g^{*}+W^{g\to p}g_{t}\in R^{d_{p}}$, where $g^{*}$ and $g_{t}$ are the goal and current GC patterns, while $W^{in}$ and $W^{g\to p}$ are the input and GC-to-planner projection matrices. This combined input has the same dimension as the planner network. Conceptually, it can be interpreted as the planning vector $v^{\text{plan}}$, while the planner’s recurrent matrix represents the transition matrix T. The resulting connectivity matrix is shown in Figure 3f.

During training, we generate random 1-second trajectories to sample pairs of current and target locations, allowing the animal to learn local transition rules. These trajectories are used only to ensure that the target location is reachable within 1 second from the current location; the trajectories themselves are not provided to the planning subnetwork. The planning subnetwork is trained to minimize the mean squared error between the current and target GC states for all timesteps.

For testing, we generate longer 10 and 20 second trajectories to define start and target locations, again without providing the full trajectories to the planner. The GC states produced during planning are decoded at each step to infer the locations the animal is virtually planned to reach. As shown in Figure 3g, the dots represent these decoded locations along the planned path, while the colored line shows the full generated trajectory for visualization and comparison. We observe that the planner generalizes from local to long-range planning and can take paths that shortcut the trajectories used to generate start and target locations. Notably, even when trained on just 128 fixed start-end pairs over 100 steps, it still successfully plans paths between locations reachable over 10 seconds.

#### Training the Planning Network Together with HC-MEC Enables Reconstruction of Sensory Experiences Along Planned Paths

We next test whether the HC-MEC loop enables the network to reconstruct sensory experiences along planned trajectories using intermediate GC states. To this end, we train the planning subnetwork together with the entire HC-MEC model that was pre-trained to encode the room during random traversal ($r_{\text{mask}}=1.0$, see Suppl. 5.2). We fix all projections from SMCs and HPCs to the planner to zero. This ensures that the planner uses only GC representations for planning and controls HC-MEC dynamics by producing inputs to speed and direction cells. SMCs and GCs are initialized to their respective ground truth responses at the start location.

Using the same testing procedures as before, we sampled the SMC and GC responses while the planner generated paths between two locations reachable within 10 seconds. We decoded GC and SMC activity at each timestep to locations using nearest neighbor search on their respective ratemaps. We found that the decoded SMC trajectories closely followed those of the GCs, suggesting that SMC responses can be reconstructed from intermediate GC states via HPCs (see Figure 3h). Additionally, compared to the GC-only planning case, we reduced the number of GCs in the HC-MEC model to avoid an overly large network, which would make the HC-MEC training hard to converge. Although this resulted in a less smooth GC-decoded trajectory than in Figure 3g, the trajectory decoded from SMCs was noticeably smoother. We suggest this is due to the auto-associative role of HPCs, which use relayed GC responses to reconstruct SMCs, effectively smoothing the trajectory.

#### Testing REMI for Realistic Navigation Task in Habitat Sim.

Finally, we tested whether the proposed framework generalizes to realistic navigation tasks and whether the reconstructed sensory embeddings not only correspond to the correct locations along the planned path but also retain sufficient information to recover the original sensory observations. To this end, we replaced the simulated SMC rate maps $R_{\text{smc}}\in R^{L_{x}\times L_{y}\times N}$ with visual feature embeddings extracted from the Habitat Synthetic Scenes Dataset (HSSD) [66] in the photorealistic simulator Habitat-Sim [45–47]. The new SMC rate map collected in Habitat-Sim is $R_{\text{hab}}\in R_{+}^{L_{x}\times L_{y}\times 1024}$.

**Figure 4:**
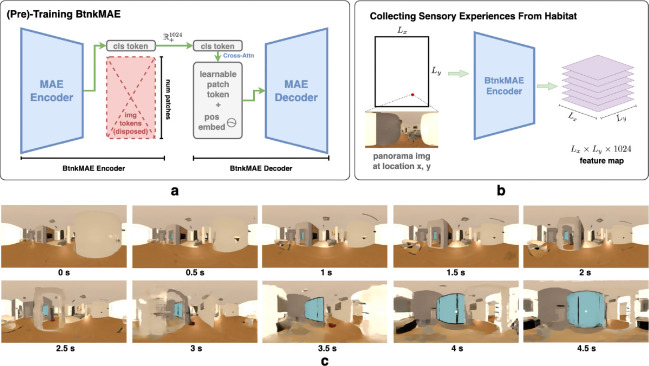
**(a)** (Pre-)training process of the BtnkMAE that uses a modification of the Masked AutoEncoder in [48]. Image features are ignored, and only the CLS token is passed to the decoder. The decoder cross-attends to the CLS token with learnable embeddings to reconstruct the image tokens such that each image can be faithfully encoded into 1024 dimensional features. See Suppl. 7. **(b)** Collection of SMC rate maps from Habitat Sim. **(c)** Decoding intermediate SMC states during path planning yields images that closely match the expected views along the planned route.

To construct $R_{\text{hab}}$, we captured panoramic images I at each spatial location with a fixed orientation and encoded them using a vision encoder that produced 1024-dimensional feature vectors $E(I)\in R_{+}^{1024}$. We additionally needed a decoder D(E(I))≈I to transform the encoded image back to the pixel space. For this purpose, we modified a pretrained Masked Autoencoder (MAE) [48] with a ViT-Large backbone, referred to as BtnkMAE. The original MAE produced image features of size $R^{p\times d}$, where p is the number of patches and d the embedding dimension, which is unsuitable for our HC-MEC model that requires a single vector representation per image. To address this, we pretrained BtnkMAE on ImageNet-1k, retaining only a retrained CLS token after the encoder to represent each image before decoding. The decoder then employed DETR-style cross-attention [67] to reconstruct the image from this single embedding vector (Figure 4a; see also Suppl. 7 for details).

We then repeated the experiment from the previous section with an additional layer normalization to stabilize training. During planning, the HC-MEC updated the SMC region to intermediate states $z_{0},\ldots ,z_{T}$, where $z_{i}\in R_{+}^{1024}$ denotes the population firing statistics of the SMC subregion. Decoding them with D yielded images $I_{0},\ldots ,I_{T}$ that closely resembled the expected views at the corresponding planned locations (Figure 4c).

## 5 Discussion

Decades of theoretical and computational studies have explored how hippocampal place cells (HPCs) and grid cells (GCs) arise, addressing the question “What do they encode?” An equally important question is “Why does the brain encode?” We suggest that place and grid representations evolved to support internally driven navigation toward vital resources such as food, water, and shelter. Thus, we ask: *given their known phenomenology, how might HPCs and GCs be wired to support internally driven navigation?*

Previous studies show that HPCs are linked to memory and context but exhibit localized spatial maps that lack the relational structure required for planning, while the periodic lattice of GCs supports path planning and generalization yet exhibits weak contextual tuning that limits direct recall from sensory cues. We propose that GCs and spatially modulated cells (SMCs) form parallel localization systems encoding complementary aspects of space: GCs provide metric structure and SMCs reflect sensory observations. HPCs link these systems through auto-association to enable bidirectional pattern completion. We built a single-layer RNN containing GCs, HPCs, SMCs, and a planning subnetwork to test whether this coupling enables three key capabilities: (1) recall of GC patterns from contextual cues, (2) generalizable GC-based planning to novel routes, and (3) reconstruction of sensory experiences along planned paths, eliminating the need for direct planning on HPC or SMC manifolds.

Our model is flexible and accommodates existing theories. In REMI, HPCs must predict both GC and SMC responses at the next timestep to pattern-complete. Since HPCs are direction-agnostic, accurately reconstructing GC and SMC activity requires encoding possible state transitions. Because adjacent locations tend to have higher transition probabilities [39], our model implicitly reflects the SR framework of HPCs, in which the transition structure is embedded in the pattern-completion dynamics. This predictive nature aligns with previous theories that predictive coding in HPCs supports planning [40], but we emphasize that using GCs may be more efficient when shortcutting unvisited locations. Lastly, while we propose that HC-MEC coupling enables planning through recall and reconstruction, a parallel idea was proposed in [68], where a similar coupling was argued to support greater episodic memory capacity.

Our theory makes several testable predictions. First, sensory cues should reactivate GC activity associated with a target location, even when the animal is stationary or in a different environment. Second, during planning, HC-MEC coupling will induce “look forward” sweeps in MEC spatial representation, resembling recent experimental results [49]. Finally, if hippocampal place cells reconstruct sensory experiences during planning, disrupting MEC-to-HC projections should impair goal-directed navigation, while disrupting HC-to-MEC feedback should reduce planning accuracy by preventing animals from validating planned trajectories internally.

### Limitations:

First, existing emergence models of GCs make it difficult to precisely control GC scales and orientations [11, 17], and so to avoid the complicating analysis of the simultaneous emergence of GCs and HPCs, we supervised GC responses during training. Future work on their co-emergence could further support our proposed planning mechanism. Second, our framework does not account for boundary avoidance, which would require extending the HC-MEC model to include boundary cell representations [35, 69, 70]. Finally, our discussion of planning with GCs assumes the environment is of similar scale to the largest grid scale. One possibility is long-range planning may involve other brain regions [65], as suggested by observations that bats exhibit only local 3D grid lattices without a global structure [64]. Animals might use GCs for mid-range navigation, while global planning stitches together local displacement maps from GC activity.

## Supplementary Material

Supplement 1
