# Supplementary material for: REMI: Reconstructing Episodic Memory During Intrinsic Path Planning
Source: bioRxiv. 2025 Jul 3:2025.07.02.662824. Preprint. [Version 1] doi: 10.1101/2025.07.02.662824 (PMC12236589; doi:10.1101/2025.07.02.662824)
Supplement: Supplementary file 1 [file NIHPP2025.07.02.662824v1-supplement-1.pdf]

bioRxiv preprint doi: https://doi.org/10.1101/2025.07.02.662824; this version posted July 3, 2025. The copyright holder for this preprint (which was not certified by peer review) is the author/funder, who has granted bioRxiv a license to display the preprint in perpetuity. It is made available under aCC-BY 4.0 International license.

1 Supplementary Materials

1.1 Condition for Positivity of Prediction Error

Given two locations, the simplest navigation task can be framed as decoding the displacement vector between them from their corresponding grid cell representations. As suggested in Section 4.3, we propose that it is sufficient for navigation even if we first decode a displacement vector within each grid module and then combine them through simple averaging. Here, we examine what conditions are required to guarantee that the resulting averaged displacement always moves the animal closer to the target.

In the 1D case, or along one axis of a 2D case, the decoded displacement vector through simple averaging is given by:

$$\hat{d} = \frac{\ell_0}{2\pi m} \cdot \left(\sum_{i=1}^{k} s^i \cdot Z_i + \sum_{i=k+1}^{m} s^i \cdot \Delta\phi_i \right)$$

After taking this decoded displacement, the remaining distance to the target along the decoded direction is $d - \hat{d}$. To ensure the animal always moves closer to the target along this axis, it suffices to show that $d - \hat{d} < d$. Which is satisfied if $m > k + \frac{1-s^{-k}}{s-1}$.

Proof. Assume that all decodable scales yield the correct displacement vectors, i.e., for all $i \in \{k+1, \cdots, m\}$, we have:

$$\frac{\ell_0 \cdot s^i \cdot \Delta\phi_i}{2\pi} = d$$

Substituting into the expression for $\hat{d}$:

$$d - \hat{d} = \frac{k}{m} \cdot d - \frac{\ell_0}{m \cdot 2\pi} \cdot \left(\sum_{i=1}^{k} s^i \cdot Z_i \right)$$

And thus we require

$$d - \hat{d} < d \quad \Leftrightarrow \quad (k-m) \cdot d < \frac{\ell_0}{2\pi} \cdot \left(\sum_{i=1}^{k} s^i \cdot Z_i \right)$$

Since that k is the index that delineates the decodable and undercovered scales, $(\ell_0 \cdot s^k)/2 \le d < (\ell_0 \cdot s^{k+1})/2$. The worst case occurs when d takes its maximum value $(\ell_0 \cdot s^{k+1})/2$ while Z_i takes its minimum value $-\pi$. Substituting these:

$$(k-m) \cdot \frac{\ell_0 \cdot s^{k+1}}{2} < \frac{\ell_0}{2\pi} \cdot \left(\sum_{i=1}^{k} s \cdot (-\pi) \right)$$

$$\Rightarrow (k-m) \cdot s^{k+1} < -\sum_{i=1}^{k} s^i = -\frac{s(s^k-1)}{s-1}$$

$$\Rightarrow k - m < -\frac{s(s^k-1)}{s^k s(s-1)} = -\frac{1-s^{-k}}{s-1}$$

$$\Rightarrow m > k + \frac{1-s^{-k}}{s-1}$$

Notice that this bound only extend the initial assumption $m > k$ by $\frac{1-s^{-k}}{s-1}$ which never exceeds 1 when $s = e$ for 1D case, and never exceeds 2 when $s = \sqrt{e}$ for 2D space. Therefore, simple averaging reliably decreases the distance to the goal if $m > k$ in 1D and $m > k+1$ in 2D.

□

bioRxiv preprint doi: https://doi.org/10.1101/2025.07.02.662824; this version posted July 3, 2025. The copyright holder for this preprint (which was not certified by peer review) is the author/funder, who has granted bioRxiv a license to display the preprint in perpetuity. It is made available under aCC-BY 4.0 International license.

2 Metrics

2.1 Spatial Information Content

We use spatial information content (SIC) [73] to measure the extent to which a cell might be a place cell. The SIC score quantifies how much knowing the neuron's firing rate reduces uncertainty about the animal's location. The SIC is calculated as

$$I = \sum_{i}^{N} p_i \cdot \frac{r_i}{\mathbb{E}[r]} \cdot \log_2 \left(\frac{r_i}{\mathbb{E}[r]} \right)$$

Where $\mathbb{E}[r]$ is the mean firing rate of the cell, r_i is the firing rate at spatial bin i, and p_i is the empirical probability of the animal being in spatial bin i. For all cells with a mean firing rate above 0.01Hz, we discretize their firing ratemaps into 20 pixel $\times$ 20 pixel spatial bins and compute their SIC. We define a cell to be a place cell if its SIC exceeds 20.

3 Simulating Spatial Navigation Cells and Random Traversal Behaviors

3.1 Simulating Spatial Navigation Cells

In our model, we simulated the ground truth response of MEC cell types during navigation to supervise the training. We note that firing statistics for these cells vary significantly across species, environments, and experimental setups. Moreover, many experimental studies emphasize the phenomenology of these cell types rather than their precise firing rates. Thus, we simulate each type based on its observed phenomenology and scale its firing rate using the most commonly reported values in rodents. This scaling does not affect network robustness or alter the conclusions presented in the main paper. However, the relative magnitudes of different cell types can influence training dynamics. To mitigate this, we additionally employ a unitless loss function that ensures all supervised and partially supervised units are equally emphasized in the loss (see Suppl. 5.3.3).

Spatial Modulated Cells: We generate spatially modulated cells following the method in [16]. Notably, the simulated SMCs resemble the cue cells described in [28]. To construct them, we first generate Gaussian white noise across all locations in the environment. A 2D Gaussian filter is then applied to produce spatially smoothed firing rate maps.

Formally, let $\mathcal{A} \subset \mathbb{R}^2$ denote the spatial environment, discretized into a grid of size $W \times H$. For each neuron i and location $\mathbf{x} \in \mathcal{A}$, the initial response is sampled as i.i.d. Gaussian white noise: $\epsilon_i(\mathbf{x}) \sim \mathcal{N}(0, 1)$. Each noise map ϵ_i is then smoothed via 2D convolution with an isotropic Gaussian kernel G_{σ_i}, where σ_i represents the spatial tuning width of cell i. The raw cell response is then given by:

$$R_i^{\text{raw}} = \epsilon_i * G_{\sigma_i}$$

where $*$ denotes the 2D convolution. The spatial width σ_i is sampled independently for each cell using $\mathcal{N}(12\text{cm}, 3\text{cm})$. Finally, the response of each cell is normalized using min-max normalization:

$$R_i = \frac{R_i^{\text{raw}} - \min(R_i^{\text{raw}})}{\max(R_i^{\text{raw}}) - \min(R_i^{\text{raw}})}$$

The SMCs used in our experiments model the sensory-related responses and non-grid cells in the MEC. Cue cells reported in [28] typically exhibit maximum firing rates ranging from 0–20Hz, but show lower firing rates at most locations distant from the cue. Non-grid cells reported in [74] generally have peak firing rates between 0–15Hz. To align with these experimental observations, we scale all simulated SMCs to have a maximum firing rate of 15Hz.

Grid Cells: To simulate grid cells, we generate each module independently. For a module with a given spatial scale ℓ, we define two non-collinear basis vectors

$$\mathbf{b}_1 = \begin{bmatrix} \ell \\ 0 \end{bmatrix} \quad \text{and} \quad \mathbf{b}_2 = \begin{bmatrix} \ell/2 \\ \ell\sqrt{3}/2 \end{bmatrix}$$

These vectors generate a regular triangular lattice:

$$\mathcal{C} = \{ n\mathbf{b}_1 + m\mathbf{b}_2 \mid n, m \in \mathbb{Z} \}$$

bioRxiv preprint doi: https://doi.org/10.1101/2025.07.02.662824; this version posted July 3, 2025. The copyright holder for this preprint (which was not certified by peer review) is the author/funder, who has granted bioRxiv a license to display the preprint in perpetuity. It is made available under aCC-BY 4.0 International license.

For each module, we randomly pick its relative orientation with respect to the spatial environment by selecting a random $\theta \in [0, \pi/3)$ which is used to rotate the lattice:

$$\mathbf{R}^\theta = \begin{bmatrix} \cos\theta & -\sin\theta \\ \sin\theta & \cos\theta \end{bmatrix}, \quad \mathcal{C}_\theta = \{\mathbf{R}^\theta \mathbf{c} \mid \mathbf{c} \in \mathcal{C}\}$$

Within each module, individual cells are assigned unique spatial phase offsets. These offsets $\boldsymbol{\psi}_i$ are sampled from a equilateral triangle with vertices

$$V_1 = \mathbf{R}^\theta \begin{bmatrix} 0 \\ 0 \end{bmatrix} \quad V_2 = \mathbf{R}^\theta \begin{bmatrix} 0 \\ -\ell/2 \end{bmatrix} \quad V_3 = \mathbf{R}^\theta \begin{bmatrix} \ell\sqrt{3}/2 \\ -\ell/2 \end{bmatrix}.$$

We sample phase offsets for grid cells within each module by drawing vectors uniformly from the triangular region using the triangle reflection method. Since the resulting grid patterns are wrapped around the lattice, this is functionally equivalent to sampling uniformly from the full parallelogram. The firing centers for cell i in a given module are then given by:

$$\mathcal{C}_i = \{\mathbf{c}_i^* + \boldsymbol{\psi}_i \mid \mathbf{c}_i^* \in \mathcal{C}_\theta\}$$

Finally, the raw firing rate map for cell i is generated by placing isotropic Gaussian bumps centered at each location in $\mathcal{C}_i$:

$$R_i^{\text{raw}} = \sum_{\mathbf{c}_i \in \mathcal{C}_i} \exp\left(-\frac{\|\mathbf{x} - \mathbf{c}_i\|^2}{2\sigma_{\text{grid}}^2}\right), \quad \mathbf{x} \in \mathcal{A}$$

where σ_{grid} is the spatial tuning width of each bump and is computed as: $2\sigma_{\text{grid}} = \ell/r$ with $r = 3.26$ following the grid spacing to field size ratio reported in [59]. Experimental studies have reported that rodent grid cells typically exhibit maximum firing rates in the range of 0–15Hz [7, 58], though most observed values are below 10Hz. Accordingly, we scale the generated grid cells to have a maximum firing rate of 10Hz.

Speed Cells: Many cells in the MEC respond to speed, including grid cells, head direction cells, conjunctive cells, and uncategorized cells [75, 76]. These cells may show saturating, non-monotonic, or even negatively correlated responses to movement speed. To maintain simplicity in our model, we represent speed cells as units that respond exclusively and linearly to the animal's movement speed. Specifically, we simulate these cells with a linear firing rate tuning based on the norm of the displacement vector $\vec{d}$ at each time step, which reflects the animal's instantaneous speed.

Additionally, many reported speed cells exhibit high baseline firing rates [75, 76]. To avoid introducing an additional parameter, we set all speed cells' tuning to start from zero—i.e., their firing rate is 0Hz when the animal is stationary. To introduce variability across cells, each speed cell is assigned a scaling factor s_i sampled from $\mathcal{N}(0.2, 0.05)$ Hz/(cm/s), allowing cells to fire at different rates for the same speed input. Given that the simulated agent has a mean speed of 10cm/s (Suppl. 3.2), the average firing rate of speed cells is approximately 2Hz. We chose a lower mean firing rate than observed in rodents, as we did not include a base firing rate for the speed cells. However, this scaling allows cells with the strongest speed tuning to reach firing rates up to 80Hz, matching the peak rates reported in [76] when the agent moves at its fastest speed.

All cells follow the same linear speed tuning function:

$$R_i(\vec{d}) = \frac{s_i \cdot \|\vec{d}\|}{dt}$$

where dt is the simulation time resolution (in seconds), $\|\vec{d}\|$ is the displacement magnitude over that interval, and s_i modulates the response sensitivity of each cell.

Direction Cells: We define direction cells based on the movement direction of the animal, rather than its body or head orientation. During initialization, each cell is assigned a preferred allocentric direction drawn uniformly at random from the interval $[0, 2\pi)$. At any time during the animal's movement, we extract its absolute displacement vector $\vec{d}$. From this vector, we compute the angular difference $\Delta\theta$ between the allocentric movement direction and the i-th cell's preferred direction. The movement direction can be derived from the displacement vector $\vec{d}$. Each neuron responds according to a wrapped Gaussian tuning curve:

$$R_i(\theta) = \exp\left(-\frac{[\Delta\theta_i]_{2\pi}^2}{2\sigma_{\text{dir}}^2}\right)$$

bioRxiv preprint doi: https://doi.org/10.1101/2025.07.02.662824; this version posted July 3, 2025. The copyright holder for this preprint (which was not certified by peer review) is the author/funder, who has granted bioRxiv a license to display the preprint in perpetuity. It is made available under aCC-BY 4.0 International license.

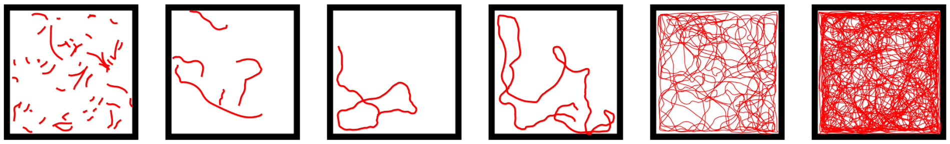

Figure S1: Example generated trajectories of varying lengths. From left to right: 50 trajectories of 1s each, 5 trajectories of 5s, followed by single trajectories of 30s, 1min, 10min, and 30min.

where $[\Delta\theta_i]_{2\pi}$ denotes the angular difference wrapped into the interval $[0, 2\pi)$. σ_{dir} denotes the tuning width (standard deviation) of the angular response curve. We set $\sigma_{\text{dir}} = 1$ rad to reduce the number of direction cells needed to span the full angular space $[0, 2\pi)$, thereby decreasing the size of the RNN to improve training efficiency. Given that many head direction cells in the MEC are conjunctive with grid and speed cells [77], we set the mean firing rate of direction cells to 2Hz to match the typical firing rates of speed cells.

We acknowledge that this simulation of the direction cell may only serve as a simplified model. However, in our model, direction cells serve only to provide input to grid cells for path integration, and we have verified that the precise tuning width and magnitude do not affect the planning performance or change the conclusion of the main text. Our findings could be further validated by future studies employing more biologically realistic simulation methods.

3.2 Simulating Random Traversal Behaviors

We train our HC-MEC model using simulated trajectories of random traversal, during which we sample masked observations of displacement and sensory input. The network is trained to reconstruct the unmasked values of speed, direction, SMC, and grid cell responses at all timepoints. Trajectories are simulated within a $100 \times 100\text{cm}^2$ arena, discretized at 1 cm resolution per spatial bin. The agent's movement is designed to approximate realistic rodent behavior by incorporating random traversal with momentum.

At each timestep, the simulated agent's displacement vector $\vec{d}$ is determined by its current velocity magnitude, movement direction, and a stochastic drift component. The base velocity v is sampled from a log-normal distribution with mean $\mu_{\text{spd}} = 10$ and standard deviation $\sigma_{\text{spd}} = 10$ (in cm/s), such that the agent spends most of its time moving below 10 cm/s but can reach up to 50 cm/s. The velocity is converted to displacement by dividing by the simulation time resolution dt, and re-sampled at each timestep with a small probability p_{spd} to introduce variability.

To simulate movement with momentum, we add a drift component that perturbs the agent's displacement. The drift vector is computed by sampling a random direction, scaling it by the current velocity and a drift coefficient c_{drift} that determines the drifting speed. Drift direction is resampled at each step with a small probability p_{dir} to simulate the animal switch their traversal direction. The drift is added to the direction-based displacement, allowing the agent to move in directions slightly offset from its previous heading. This results in smooth trajectories that preserve recent movement while enabling gradual turns.

To prevent frequent collisions with the environment boundary, a soft boundary-avoidance mechanism is applied. When the agent is within d_{avoid} pixels of a wall and its perpendicular distance to the wall is decreasing, an angular adjustment is applied to its direction. This correction is proportional to proximity and only engages when the agent is actively moving toward the wall. We set $dt = 0.01$ sec/timestep, $p_{\text{spd}} = 0.02$, $c_{\text{drift}} = 0.05$, $p_{\text{dir}} = 0.15$, and $d_{\text{avoid}} = 10$ pixels. These values were chosen to produce trajectories that qualitatively match rodent traversal (see Fig S1).

4 Decoding Location from Population Activity

In the main text, we decode the population vector to locations in both the recall task (Section 3) and the planning task (Section 4.4). Here we present the method we used for such decoding. Given a population vector $\mathbf{r} \in \mathbb{R}^N$ at a given timestep, we decode it into a location estimate by performing nearest neighbor search over a set of rate maps corresponding to the subpopulation of

bioRxiv preprint doi: https://doi.org/10.1101/2025.07.02.662824; this version posted July 3, 2025. The copyright holder for this preprint (which was not certified by peer review) is the author/funder, who has granted bioRxiv a license to display the preprint in perpetuity. It is made available under aCC-BY 4.0 International license.

cells corresponding to $\mathbf{r}$. These rate maps may come from our simulated ground-truth responses or be aggregated from the network's activity during testing (see Suppl. 5.4).

Formally, let $\mathbf{r} = [r_1, r_2, \cdots, r_N]$ be the population response of a subpopulation of N cells at a given timestep, and let $M \in \mathbb{R}^{P \times N}$ be the flattened rate maps, where each row m_p corresponds to the population response at the p-th spatial bin. Here, $P = W \times H$ is the total number of discretized spatial bins in the environment $\mathcal{A}$. The decoding process is to find the index p^* that minimizes the Euclidean distance between $\mathbf{r}$ and m_p:

$$p^* = \arg\min_p \|\mathbf{r} - m_p\|_2$$

To efficiently implement this decoding, we use the FAISS library [60, 61]. Specifically, we employ the `IndexIVFFlat` structure, which first clusters the rows $\{m_p\}_{p=1}^P$ into k clusters using k-means. Each vector m_p is then assigned to its nearest centroid, creating an inverted index that maps each cluster to the set of vectors it contains.

At query time, the input vector $\mathbf{r}$ is first compared to all centroids to find the `n_probe` closest clusters. The search is then restricted to the vectors assigned to these clusters. Finally, the nearest neighbor among them is returned, and its index p^* is mapped back to the corresponding spatial coordinate $\mathbf{x}^*$. For all experiments, we set the `n_clusters` for the k-means to 100 and `n_probe` to 10.

5 Training and Testing of the HC-MEC Model

As described in Section 2.1, our HC-MEC model is a single-layer RNN composed of multiple sub-networks. We train two versions of this model: (1) **GC-only** variant, which includes only grid cells and along with speed and direction cells; and (2) the full **HC-MEC** loop model, which includes both MEC and hippocampal (HC) subpopulations.

In both cases, we simulate ground-truth responses for supervised and partially supervised cells using the method described in Suppl. 3. The number of simulated cells matches exactly the number of corresponding units in the RNN hidden layer. That is, if we simulate N_g grid cells, we assign precisely N_g hidden units in the RNN to represent them, and (partially)-supervise these units with the corresponding ground-truth activity. Additional details on this partial supervision are provided in Suppl. 5.1.

For both models, we use six scales of grid cells, with the initial spatial scale set to 30cm. Subsequent scales follow the theoretically optimal scaling factor $s = \sqrt{e}$ [12]. The grid spacing to field size ratio is fixed at 3.26 [59]. Each scale comprises 48 grid cells, and the spatial phase offsets for each cell within a module are independently sampled from the corresponding equilateral triangle (Suppl. 3) with side length equals to the spatial scale of the module. In addition, both the **GC-only** and **HC-MEC** models include 32 speed cells and 32 direction cells.

The **HC-MEC** model additionally includes spatially modulated cells (SMCs) in the MEC subpopulation and hippocampal place cells (HPCs) in the HC subpopulation. We include 256 SMCs to approximately match the number of grid cells. These SMCs are designed to reflect responses to sensory experience and are trained with supervision as described in Suppl. 3. We also include 512 HPCs, matching approximately the total number of grid cells and SMCs (N = 256 + 288). These cells only receive input from and project to the MEC subpopulation through recurrent connections, and thus do not receive any external input. We note that, differing from [16], we did not apply a firing rate constraint on the HPCs, but still observed the emergence of place cell-like responses.

In total, unless otherwise specified, our **GC-only** model comprises 352 hidden units (48×6 grid cells $+$ 32 speed cells $+$ 32 direction cells), while the **HC-MEC** model comprises 1120 hidden units (288 grid cells $+$ 32 speed cells $+$ 32 direction cells $+$ 256 SMCs $+$ 512 HPCs).

5.1 Supervising Grid Cells

As described in Section 2.1, we partially supervise the grid cell subpopulation of the RNN using simulated ground-truth responses. Let $\mathbf{z}_t \in \mathbb{R}^{N_g}$ denote the hidden state of the RNN units modeling grid cells at time t. During training, the HC-MEC model is trained on short random traversal trajectories. Along each trajectory, we sample the ground-truth grid cell responses from the simulated ratemaps at the corresponding location and denote these as $\{\mathbf{r}_t^g\}_{t=0}^T$. At the start of each trajectory, we initialize the grid cell units with the ground-truth response at the starting location, i.e., $\mathbf{z}_0 = \mathbf{r}_0^g$.

bioRxiv preprint doi: https://doi.org/10.1101/2025.07.02.662824; this version posted July 3, 2025. The copyright holder for this preprint (which was not certified by peer review) is the author/funder, who has granted bioRxiv a license to display the preprint in perpetuity. It is made available under aCC-BY 4.0 International license.

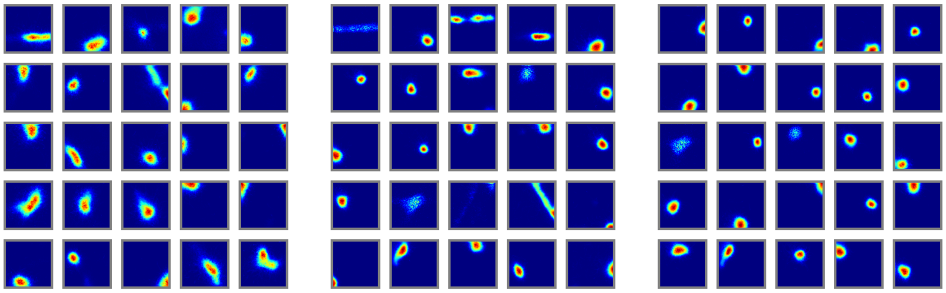

Figure S2: Example place fields emerging over training. Shown are place fields from epoch 2, 20, and 50. As training progresses, the fields become increasingly specific and spatially refined.

From time step $t = 1$ to T, the grid cell hidden states $\mathbf{z}_t$ are updated solely through recurrent projections between the grid cell subpopulation and the speed and direction cells. They do not receive any external inputs. After the RNN processes the speed and direction inputs over the entire trajectory, we collect the hidden states of the grid cell subpopulation $\{\mathbf{z}_t\}_{t=1}^{T}$ and minimize their deviation from the corresponding ground-truth responses $\{\mathbf{r}_t^g\}_{t=1}^{T}$. The training loss function is described in Suppl. 5.3.3.

We choose to partially supervise the grid cells due to their critical role in the subsequent training of the planning network. This supervision allows the model to learn stable grid cell patterns, reducing the risk of instability propagating into later stages of training. While this partial supervision does not reflect fully unsupervised emergence, it can still be interpreted as a biologically plausible scenario in which grid cells and place cells iteratively refine each other's representations. Experimentally, place cell firing fields appear before grid cells but become more spatially specific as grid cell patterns stabilize, potentially due to feedforward projections from grid cells to place cells [56, 57].

We observe a similar phenomenon during training. Even with partial supervision, grid cells tend to emerge after place cells. This may be because auto-associating spatially modulated sensory representations is easier than learning the more structured path-integration task hypothesized to be performed by grid cells. After the grid cells' pattern stabilizes, we also observe that place cell firing fields become more refined (Fig. S2), consistent with experimental findings [3, 54–56].

5.2 HC-MEC Training Task

During navigation, animals may use two different types of sensory information to help localize themselves: (1) sensory input from the environment to directly observe their location; and (2) displacement information from the previous location to infer the current location and potentially reconstruct the expected sensory observation. We posit that the first type of information is reflected by the weakly spatially modulated cells (SMCs) in the MEC, while the second type is reflected by grid cells and emerges through path integration.

However, as we previously argued in Section 2.1, both types of information are subject to failure during navigation. Decades of research have revealed the strong pattern completion capabilities of hippocampal place cells. We thus hypothesize that hippocampal place cells may help reconstruct one type of representation from the other through auto-associative mechanisms.

To test this hypothesis, we simulate random traversal trajectories and train our **HC-MEC** model with masked sensory inputs. The network is tasked with reconstructing the ground-truth responses of all MEC subpopulations from simulation, given only the masked sensory input along the trajectory. Specifically, the supervised units—SMCs, speed cells, and direction cells—receive masked inputs to simulate noisy sensory perception. We additionally mask the ground-truth responses used to initialize both supervised and partially supervised units at $t = 0$, so that the network dynamics also begin from imperfect internal states.

To simulate partial or noisy observations, we apply masking on a per-trajectory and per-cell basis. For a given trajectory, we sample the ground-truth responses along the path to form a matrix

bioRxiv preprint doi: https://doi.org/10.1101/2025.07.02.662824; this version posted July 3, 2025. The copyright holder for this preprint (which was not certified by peer review) is the author/funder, who has granted bioRxiv a license to display the preprint in perpetuity. It is made available under aCC-BY 4.0 International license.

$\mathbf{R} = [\mathbf{r}_0 \cdots \mathbf{r}_T]^\top \in \mathbb{R}^{T\times N}$, where N is the number of cells in the sampled subpopulation and $\mathbf{r}_t$ is the ground-truth population response at time t. The masking ratio r_{mask} defines the maximum fraction of sensory and movement-related inputs, as well as initial hidden states, that are randomly zeroed during training to simulate partial or degraded observations. We generate a binary mask $\mathbf{M} \in \{0,1\}^{T\times N}$ by thresholding a matrix of random values drawn uniformly from $[0,1]$, such that approximately $100 \times r_{\text{mask}}$ percent of the entries are set to zero. The final masked response is then obtained by elementwise multiplication $\tilde{\mathbf{R}} = \mathbf{R} \odot \mathbf{M}$.

During training, we sample multiple trajectories to form a batch, with each trajectory potentially having a different masking ratio and masked positions. For both the HC-MEC model used in the recall task (Section 2) and the one pre-trained for the planning task (Section 4.4), masking ratios for SMCs and other inputs are sampled independently from the interval $[0, r_{\text{mask}}]$. Specifically, for each trial, we sample:

$$m_{\text{SMC}}, m_{\text{other}} \sim \mathcal{U}(0, r_{\text{mask}})$$

We use m_{SMC} to generate the mask for SMC cells, and m_{other} to independently generate masks for grid cells, speed cells, and direction cells. This allows the model to encounter a wide range of noise conditions during training—for example, scenarios where sensory inputs are unreliable but displacement-related cues are available, and vice versa.

5.3 RNN Implementation

5.3.1 Initialization

Our HC-MEC model includes multiple sub-regions. However, we aim to model these sub-regions without imposing explicit assumptions about their connectivity, as the precise connectivity—particularly the functional connectivity between the hippocampus (HC) and medial entorhinal cortex (MEC)—remains unknown. Modeling these sub-regions with multiple hidden layers would implicitly enforce a uni-directional flow of information: the second layer would receive input from the first but would not project back. Specifically, a multi-layer RNN with two recurrent layers can be represented by a block-structured recurrent weight matrix:

$$\mathbf{W} = \begin{bmatrix} \mathbf{W}^{11} & \mathbf{W}^{12} \\ \mathbf{W}^{21} & \mathbf{W}^{22} \end{bmatrix}$$

Where $\mathbf{W}^{11}$ and $\mathbf{W}^{22}$ are the recurrent weight matrices of the first and second recurrent layers, respectively, and $\mathbf{W}^{12}$ is the projection weights from the first to the second layer. In typical multi-layer RNN setups, $\mathbf{W}^{12} = \mathbf{0}$, meaning that the second sub-region does not send information back to the first. This structure generalizes to deeper RNNs, where only the diagonal blocks $\mathbf{W}^{ii}$ and the blocks directly below the diagonal $\mathbf{W}^{(i+1)i}$ are non-zero.

Therefore, we model the HC-MEC system as a large single-layer RNN, such that all sub-blocks are initialized as non-zero, and their precise connectivity is learned during training and entirely defined by the task. To initialize this block-structured weight matrix, we first initialize each subregion independently as a single-layer RNN with a defined weight matrix but no active dynamics. Suppose we are modeling N_r subregions, and each subregion i contains d_i hidden units. We initialize the recurrent weights within each subregion using a uniform distribution:

$$\mathbf{W}^{ii} \sim \mathcal{U}\left(-1/\sqrt{d_i}, 1/\sqrt{d_i}\right)$$

For each off-diagonal block $\mathbf{W}^{ij}$, corresponding to projections from subregion j to subregion i, we similarly initialize:

$$\mathbf{W}^{ij} \sim \mathcal{U}\left(-1/\sqrt{d_j}, 1/\sqrt{d_j}\right)$$

Note that the initialization bound is determined by the size of the source subregion j, consistent with standard practices for stabilizing the variance of the incoming signals. Once initialized, all sub-blocks are copied into their respective locations within a full recurrent weight matrix:

$$\mathbf{W}_{\text{HCMEC}} = \begin{bmatrix} \mathbf{W}^{11} & \cdots & \mathbf{W}^{1N} \\ \vdots & \ddots & \vdots \\ \mathbf{W}^{N_r 1} & \cdots & \mathbf{W}^{N_r N_r} \end{bmatrix}$$

bioRxiv preprint doi: https://doi.org/10.1101/2025.07.02.662824; this version posted July 3, 2025. The copyright holder for this preprint (which was not certified by peer review) is the author/funder, who has granted bioRxiv a license to display the preprint in perpetuity. It is made available under aCC-BY 4.0 International license.

with total size $\sum_{i=1}^{N_r} d_i \times \sum_{i=1}^{N_r} d_i$.

Additionally, as described in main text, both input and output neurons are already modeled within the HC-MEC model. As a result, no additional input or output projections are required. Input neurons in this assembled RNN directly integrate external signals from the simulation, while the states of output neurons are directly probed out during training.

5.3.2 Parameters

For models used in the recall and planning tasks, we use the following parameters:

Table 1: Shared parameters for **GC-only** and **HC-MEC** models

Parameter	Value	Description
N_{speed}	32	Number of speed cells
$N_{\text{direction}}$	32	Number of direction cells
N_{grid} (per module)	48	Number of grid cells per module
`n_modules`	6	Number of spatial scales (modules)
`initial_scale`	30 cm	Spatial period of the smallest module
`spatial_scale_factor`	$\sqrt{e}$	Scale ratio between adjacent modules
$r_{\text{grid/field}}$	3.26	Grid spacing to field size ratio
`activation`	ReLU	Activation function of the hidden units
dt	0.05 s	Time res for both cell simulation and RNN, 1s = 20 bins
α_{init}	0.2	Initial forgetting rate of the cells
`learn_alpha`	True	Whether the forgetting rate is learned during training
`optimizer`	AdamW	Optimizer
`learning_rate`	0.001	Learning rate
`batch_size`	128	Batch size, each batch corresponds to a single trajectory
`n_epochs`	50	Number of training epochs
`n_steps`	1000	Number of steps per trajectory
$T_{\text{trajectory}}$	2 s	Duration of each training trajectory

In Section 4.4 of the main text, we noted that the **GC-Only** model used for planning uses $N_{\text{grid}} = 128$, i.e., each module comprised 128 grid cells. This larger population improves planning accuracy, likely due to denser coverage of the space. However, for the full **HC-MEC** model used in planning, we reverted to $N_{\text{grid}} = 48$, consistent with our default configuration. As discussed in the main text, the auto-associative dynamics from place cells help smooth the trajectory, even when the decoded trajectory from grid cells is imperfect.

Table 2: Additional parameters for **HC-MEC** model

Parameter	Value	Description
N_{SMC}	256	Number of spatially modulated cells
σ_{SMC}	$\mathcal{N}(12\text{cm}, 3\text{cm})$	Width of Gaussian smoothing to generate SMCs, which controls the spatial sensitivity of SMCs (see Suppl. 3)
N_{HPC}	512	Number of hippocampal place cells

5.3.3 Loss Function

We use a unitless MSE loss for all supervised and partially supervised units such that all supervised cell types are equally emphasized. For each trajectory, we generate ground-truth responses by sampling the corresponding region's simulated ratemaps at the trajectory's locations, resulting in $\{\mathbf{r}_t\}_{t=0}^{T}$. After the RNN processes the full trajectory, we extract the hidden states of the relevant units to obtain $\{\mathbf{z}_t\}_{t=0}^{T}$. To ensure that all loss terms are optimized equally and are not influenced by the scale or variability of individual cells, we perform per-cell normalization of the responses. For each region i, we compute the mean $\boldsymbol{\mu}^i \in \mathbb{R}^{d_i}$ and standard deviation $\boldsymbol{\sigma}^i \in \mathbb{R}^{d_i}$ of the ground-truth responses across the time and batch dimensions, independently for each cell. The responses are then normalized

bioRxiv preprint doi: https://doi.org/10.1101/2025.07.02.662824; this version posted July 3, 2025. The copyright holder for this preprint (which was not certified by peer review) is the author/funder, who has granted bioRxiv a license to display the preprint in perpetuity. It is made available under aCC-BY 4.0 International license.

elementwise:

$$\hat{\mathbf{r}}_t^i = \frac{\mathbf{r}_t^i - \boldsymbol{\mu}^i}{\boldsymbol{\sigma}^i}, \quad \hat{\mathbf{z}}_t^i = \frac{\mathbf{z}_t^i - \boldsymbol{\mu}^i}{\boldsymbol{\sigma}^i}$$

The total loss across all regions is computed as the mean squared error between the normalized responses:

$$\mathcal{L} = \frac{1}{T} \sum_{i=1}^{N_r} \sum_{t=1}^{T} \lambda_i \left\| \hat{\mathbf{z}}_t^i - \hat{\mathbf{r}}_t^i \right\|_2^2$$

where λ controls the relative weights of each cell types. We set the $\lambda = 10$ for both grid cells and SMCs, while $\lambda = 1$ for velocity and direction cells. These relative weights are set as animals may emphasize their reconstruction of the sensory experience and relative location, rather than their precise speed and direction during their spatial traversal.

5.4 Constructing Ratemaps During Testing

After training, we test the agent using a procedure similar to training to estimate the firing statistics of hidden units at different spatial locations and construct their ratemaps. Specifically, we pause weight updates and generate random traversal trajectories. The supervised and partially supervised units are initialized with masked ground-truth responses, and the supervised units continue to receive masked ground-truth inputs at each timestep. We record the hidden unit activity of the RNN at every timestep and aggregate their average activity at each spatial location.

Let $\mathbf{z}_t \in \mathbb{R}^d$ be the hidden state or a subpopulation of hidden states of the RNN at time t, where d is the number of hidden units. For each unit i, the ratemap value at location $\mathbf{x}$ is computed as:

$$R_i(\mathbf{x}) = \begin{cases} \frac{1}{N(\mathbf{x})} \sum_{t:\mathbf{x}_t=\mathbf{x}} \mathbf{z}_t^i & \text{if } N(\mathbf{x}) > 0 \\ \texttt{NaN} & \text{otherwise} \end{cases}$$

where $N(\mathbf{x})$ is the number of times location $\mathbf{x}$ was visited during testing. We set the trajectory length to T = 5s (250 time steps), `batch_size` $= 512$, and `n_batches` $= 200$. We perform this extensive testing to ensure that the firing statistics of all units are well estimated. Each spatial location is visited on average 1008.13 ± 268.24 times (mean ± standard deviation).

6 Recall Task

In this paper, we have posited that the auto-association of place cells may trigger the reconstruction of grid cell representations given sensory observations. To test whether such reconstruction is possible, we trained nine different models, each with a fixed masking ratio r_{mask} (m_r in the main text) ranging from 0.1 to 0.9. Following the procedure described in Suppl. 5.2, each model was trained with the same masking ratio applied to all subregions and across all trials, but the masking positions were generated independently for each trial. That is, for each trial, $100 \times r_{\text{mask}}$ of the entries were occluded. The mask was applied both to the ground-truth responses used to initialize the network and to the subsequent inputs.

After training, we tested recall by randomly selecting a position in the arena and sampling the ground-truth response of the SMC subpopulation to represent the sensory cues. This sampled response was then repeated over $T = 10$s (200 time steps) to form a constant input query. Unlike training, the initial state of the network was set to zero across all units, such that the network dynamics evolved solely based on the queried sensory input.
